# The Multiple Roles of Hypothetical Gene BPSS1356 in *Burkholderia pseudomallei*

**DOI:** 10.1371/journal.pone.0099218

**Published:** 2014-06-13

**Authors:** Hokchai Yam, Ainihayati Abdul Rahim, Suriani Mohamad, Nor Muhammad Mahadi, Uyub Abdul Manaf, Alexander Chong Shu-Chien, Nazalan Najimudin

**Affiliations:** 1 School of Biological Sciences, Universiti Sains Malaysia, Minden, Pulau Pinang, Malaysia; 2 Faculty of Agro Based Industry, Universiti Malaysia Kelantan, Jeli, Kelantan, Malaysia; 3 School of Pharmaceutical Sciences, Universiti Sains Malaysia, Minden, Pulau Pinang, Malaysia; 4 Comparative Genomics and Genetics Research Centre, Malaysia Genome Institute, Kajang, Selangor, Malaysia; University of North Dakota School of Medicine and Health Sciences, United States of America

## Abstract

*Burkholderia pseudomallei* is an opportunistic pathogen and the causative agent of melioidosis. It is able to adapt to harsh environments and can live intracellularly in its infected hosts. In this study, identification of transcriptional factors that associate with the β′ subunit (RpoC) of RNA polymerase was performed. The N-terminal region of this subunit is known to trigger promoter melting when associated with a sigma factor. A pull-down assay using histidine-tagged *B. pseudomallei* RpoC N-terminal region as bait showed that a hypothetical protein BPSS1356 was one of the proteins bound. This hypothetical protein is conserved in all *B. pseudomallei* strains and present only in the *Burkholderia* genus. A BPSS1356 deletion mutant was generated to investigate its biological function. The mutant strain exhibited reduced biofilm formation and a lower cell density during the stationary phase of growth in LB medium. Electron microscopic analysis revealed that the ΔBPSS1356 mutant cells had a shrunken cytoplasm indicative of cell plasmolysis and a rougher surface when compared to the wild type. An RNA microarray result showed that a total of 63 genes were transcriptionally affected by the BPSS1356 deletion with fold change values of higher than 4. The expression of a group of genes encoding membrane located transporters was concurrently down-regulated in ΔBPSS1356 mutant. Amongst the affected genes, the putative ion transportation genes were the most severely suppressed. Deprivation of BPSS1356 also down-regulated the transcriptions of genes for the arginine deiminase system, glycerol metabolism, type III secretion system cluster 2, cytochrome bd oxidase and arsenic resistance. It is therefore obvious that BPSS1356 plays a multiple regulatory roles on many genes.

## Introduction

*Burkholderia pseudomallei* is an opportunistic pathogen that infect higher eukaryotes including human. It causes a life threatening disease known as melioidosis which is endemic especially in Southern Asia [1]. This Gram-negative bacterium is an environmental saprophyte that resides commonly in wet soil and stagnant water. Multiple acquisition routes and the ability to live intracellularly in its host cells including macrophages is a distinct characteristic of *B. pseudomallei* in the development of the fatal disease [2]. Resistance to canonical antibiotics, high mortality rate of infected patients and the expansion of endemic areas are amongst the major reasons why *B. pseudomallei* is receiving great attention [3].

RNA polymerase serves as the key catalytic enzyme of transcription. A functional assembly of a RNA polymerase consists four core subunits (subunit α, β, β′ and ω) for transcriptional elongation and a sigma factor for promoter recognition. The sigma factor is known to be an essential component to respond to various growth conditions or environmental stimuli. However, the network of protein-protein interaction of each subunit of bacterial RNA polymerase is a rather intricate system. In a global protein-protein network investigation, Arifuzzaman et al. (2006) [4] reported that bacterial RNA polymerase is a highly interactive enzyme. However, the biological purposes of many of these bindings are largely unknown. The study was conducted by using a pull-down assay in which all the protein baits were recombinantly produced. A similar result was observed if the native form of the protein baits were used [5].

The process of transcription in prokaryotes involves several stages. The initial step of transcription is the formation of an open promoter complex in which the promoter is melted by separating the two DNA strands in the promoter region. Young et al. (2004) [6] showed that amino acids 1 to 314 of the β′ subunit N-terminal region and amino acids 94 to 507 of the σ^A^ subunit were sufficient to robustly melt the extended −10 promoter region. These two polypeptides comprise less than one-fifth of RNA polymerase holoenzyme. This N-terminal region of the β′ subunit contains a Zn^2+^ finger domain and a coiled-coil domain. It is responsible for the initial promoter binding and σ^70^ subunit docking, respectively [7], [8].

This minimal region of β′ subunit that causes promoter melting was recombinantly produced and later used as the bait in a pull-down assay. The interacting proteins were harvested and their identities were determined using a Maldi-TOF analysis. One of the interacting proteins was identified as hypothetical protein BPSS1356 based on the *B. pseudomallei* genome [9]. An isogenic BPSS1356 deletion mutant was constructed to elucidate the biological role of BPSS1356 in *Burkholderia pseudomallei*. A comparative phenotypic characterizations as well as an RNA microarray study were conducted on the mutant and the wild type strains.

## Materials and Methods

### Production of RpoC N-terminal protein (RpoC-N) and pull-down assay

The DNA fragment encoding RpoC-N was PCR amplified from the genomic DNA of *Burkholderia pseudomallei* K96243. This N-terminal fragment contained the minimal region of RpoC required for promoter melting during transcription initiation [6]. The genome sequence of K96243 (European Molecular Biology Laboratory accession numbers BX571965 and BX571966) reported by Holden et al. (2004) [9] was referred to in the design of the primers. The sequences of the forward and reverse primers were 5′-ATAGGATCCATCGGTCTGGCCTCGCCGGAC-3′ (The underlined nucleotides represent *BamH*I recognition sequence) and 5′-TATGGTACCGACGCGCTTGCCGAGCAGGTTC -3′ (The underlined nucleotides represent *Kpn*I recognition sequence), respectively. The primers were designed to target the coding sequence of the N-terminal region of RpoC corresponding to amino acid 32 to 347 at genomic location of 3820366 to 3820363 (978 bp). PCR amplification was performed using the high fidelity KOD DNA polymerase (Novagen, USA) accordingly to the manufacturer's instruction. The PCR amplified DNA fragment coding for N-terminal region of RpoC (approximately 1 kb) was then cloned into the expression vector pQE-30 (Qiagen, Germany) using the *BamH*I and *Kpn*I restriction sites. *Escherichia coli* JM109 was used as the cloning and expression host. The resultant plasmind was named as pQE-RPOCN and its recombinant protein contained a His-tag at the N-terminus. The plasmid pQE-RPOCN was extracted and subjected to automated DNA sequencing to verify the insert.

Mid-exponential-phase cultures of *E. coli* JM109 harboring pQE-RPOCN growing in LB medium at 30°C was induced with 0.5 mM IPTG for protein production. The recombinant RpoC-N produced appeared as inclusion body. Thus, protein denaturation and refolding were performed in order to obtain soluble form by referring to protocol suggested by Young et al. (2001) [7] with modifications. The inclusion body was denatured using Urea Buffer (100 mM NaH_2_PO_4_, 8 M urea, pH 8.0). The denatured protein was subsequently subjected to dialysis against a native buffer (50 mM NaH_2_PO_4_, 100 mM NaCl, 10% glycerol, 0.1% Triton-X, pH 8.0) using SnakeSkin (Thermo Scientific, USA) dialysis tubing of 10 k MWCO. The denatured sample was first dialysed for 6 hours against a 10 time volume of native buffer at 4°C. The procedure was repeated for another 6 hours using fresh native buffer. A prolonged dialysis period of 16 hours was performed for another round of dialysis. The dialysed sample was then collected and centrifuged at 12, 000 *g* for 20 min. The supernatant which contained soluble form of RpoC-N was collected for pull-down experiment. This soluble protein was quantified using Bradford Reagent (Sigma, USA) with bovine serum albumin used as the standard.

Pull-down assay protocol that suggested by Arifuzzaman et al. (2006) [4] was referred in this study with modifications. A single colony of *B. pseudomallei* was inoculated in 5 ml of LB medium and grown overnight at 37°C. A total of 1 ml overnight culture was used as an inoculum to inoculate 100 ml of fresh LB medium. The new culture was grown until it reached an OD_600_ value of approximately 1.0. The cells were centrifuged and resuspended in 5 ml of native buffer. Cells were then lysed using sonication. The supernatant was collected after centrifugation at 12, 000 *g* for 20 min. This soluble fraction of *B. pseudomallei* was then used for a pull-down assay.

A total of 2 mg of the His-Tagged RpoC-N protein was loaded into a 200 µl bed volume of Talon resin in a free-flow 10 ml column. The resin contained embedded cobalt ion that should immobilize the His-tagged RpoC-N. The resin was then washed twice with 2 ml washing buffer (same ingredient with native buffer). The *B. pseudomallei* protein lysate (5 ml) was then loaded into the column. The resin was then washed with 2 ml of washing buffer which was actually native buffer with 5 mM imidazole. This was performed three times. Subsequently, a total of 200 µl first elution buffer (100 mM NaH_2_PO_4_, 8 M urea, pH 8.0) was loaded into the resin and gently mixed for 15 min. This first eluted fraction contained the denatured RpoC-N interactive proteins and was collected for future analysis. The second elution buffer used was the same as the first with the imidazole added (100 mM NaH_2_PO_4_, 8 M urea, 300 mM imidazole, pH 8.0). It was loaded to the column to remove RpoC-N as well as proteins that unspecifically bound to the Talon resin. A negative control was performed using a 200 µl bed volume of Talon resin and 2 mg of *B. pseudomallei* total protein as starting materials. The manufacturer's protocol of native purification was adhered to. The washing buffer without imidazole (same ingredient with native buffer) was used in the washing step and the second elution buffer was used in the elution step.

The eluted protein samples electrophoresed using 10% SDS-PAGE and were then stained with Coomassie Blue. All the protein bands of first elution were carefully excised using a clean scalpel and each of them was transferred into a microcentrifuge tubes. Peptide digestions and purifications were performed accordingly to a protocol provided by Protein and Proteomic Centre of the National University of Singapore. The complete in gel digestion and Zip-Tip purification protocols are available in web http://www.dbs.nus.edu.sg/research/facilities/ppc/index.htm. Trysin was used for peptide digestion. The digested and desalted peptides were finally analyzed using the Maldi-TOF mass spectrometry to determine their identities. This procedure was outsourced to Protein and Proteomic Centre of the National University of Singapore as well.

### Construction of *B. pseudomallei* BPSS1356 deletion mutant

The *B. pseudomallei* BPSS1356 mutant strain carrying a markerless deletion of the BPSS1356 gene was generated via homologous recombination using a non replicative plasmid pDM-4 [10]. The mutagenesis design removed a region of BPSS1356 open reading frame that encodes for amino acid position of 8 to 1104. The upstream (US) and downstream (DS) homologous regions relative to BPSS1356 ORF were PCR amplified to yield DNA fragments of 954 bp and 998 bp, respectively. They were amplified using primer pairs of 1356USF/1356USR (US fragment) and 1356DSF/1356DSR (DS fragment). The US and DS fragments were treated with the restriction enzyme *Hind*III and the ends were subsequently ligated together. The ligated sample was used as the template to amplify the fused US-DS fragment. The amplified US-DS fragment was cloned into pGEM-T vector (Promega, USA) via the TA cloning procedure to produce pGEM1356-USDS. The US-DS fragment was subsequently recloned into a SacB-based pDM-4 plasmid through *BgI*II and *Sac*I restriction sites. *E. coli* S17-1 λ*pir* was used as the cloning host. The resultant plasmid pDM4-1356 was verified by DNA sequencing from both directions using primers NQCAT and NQREV. The plasmid pDM-1356 was introduced into *B. pseudomallei* K96243 via conjugation by biparental mating with *E. coli* S17-1λ*pir* (pDM4-1356) as donor and *B. pseudomallei* K96243 as recipient. The merodiploid strains were selected on LB agar without NaCl supplemented with 150 µg/ml of chloramphenicol and 50 µg/ml of gentamicin (to kill donor *E*. *coli*). During this merodiploid stage, the non-replicative plasmid pDM4-1356 should have been integrated into the genome of *B. pseudomallei* via a single homologous recombination step. Both wild type and BPSS1356 deletion alleles should be present in this merodiploid. It is also be resistant to chloramphenicol.

The merodiploid strain was subjected to a spontaneous second stage of homologous recombination in order to generate BPSS1356 deletion mutant of *B. pseudomallei* K96243. The plasmid pDM4-1356 contained the *sacB* gene of *Bacillus subtilis* which encodes levansucrase that will synthesize levan from sucrose. The accumulation of levan or high molecular weight fructose polymer is lethal to Gram-negative host. Thus, the merodiploid strain which contained whole plasmid recombined into chromosome will be unable to grow on sucrose supplemented media. Only the cells that have been undergone a second homologous recombination to remove the *sacB* gene can survive. This event will produce either a wild type or deletion mutant depending on the location of recombination. The grown merodiploid cells were plated on the LB agar without NaCl but containing 10% sucrose. The developed colonies were subjected to PCR screening using primer pairs of 1356U-out and 1356D-out. The screening primers used were outside of the US-DS homologous region and therefore were not involved in generation of mutant. The mutant strain should produce a PCR fragment of 2.2 kb in length; the wild type strain should produce a PCR fragment of 5.5 kb. The 2.2 kb DNA fragment was subjected to DNA sequencing using primer 1356_mutSeq to verify the deletion. The sequences of the primers used in mutant construction are available in Table S1A.

### Growth pattern of wild type and ΔBPSS1356 mutant

Growth curves of wild type *B. pseudomallei* K96243 and ΔBPSS1356 mutant in LB and M9 minimal broth medium were observed. A single colony of both strains were inoculated in 25 ml of media and grown for 24 hours at 37°C with 180 rpm rotation, respectively. A fresh medium of 250 ml were separately inoculated with each culture to an optical density value (600 nm) of 0.05. The cultures were grown at 37°C at 180 rpm. Optical density of cultures at 600 nm (OD_600_) was measured at various time intervals in a spectrophotometer (U-1900 UV/Vis spectrophotometer 200V-Hitachi, Japan). Dilutions were performed when the OD_600_ value of an undiluted culture higher than 0.5. Bacterial growth for each strain was performed in triplicates until the growth curve reached a plateau. The mean values of OD_600_ were plotted into graphs.

### Electron microscopy

Scanning electron microscopy (SEM) and transmission electron microscopy (TEM) were conducted for wild type and mutant strains. The sample processing method was adapted from Glavert (1981). The bacterial cells were harvested from exponential cultures (OD_600_ 0.5) for both scanning methods.

### Biofilm formation assay

The biofilm formation assay was conducted for the wild type *B. pseudomallei* K96243 and ΔBPSS1356 mutant using LB broth and M9 minimal medium. Biofilm formation assay protocol was adapted from O'Toole & Kolter (1998) using spectrophotometric quantification. The absorbance was measured at 595 nm using a microplate reader (Model 680, Bio-Rad Laboratories, USA). The OD_595_ values of the bacterial samples were normalized against values obtained from the negative control (uninoculated media). The mean values of triplicate samples were used for this analysis.

### Global RNA microarray analysis

The RNA samples of wild type *B. pseudomallei* K96243 and ΔBPSS1356 mutant were isolated using easy-BLUE Total RNA Extraction Kit (Intron, Korea) following strictly the manufacturer's protocol. The bacterial cells were harvested when an OD_600_ value of 1.0 was reached (exponential growth phase). A total of 3 ml culture was used as started culture for every extraction. The isolated RNA samples were subjected to DNAase (Superase, Ambion) treatment according to the manufacturer's protocol. The DNAse treated RNA samples were then subjected to phenol/chloroform extraction. The volume of RNA sample was toped up to 300 µl using RNase free ddH_2_O. A total of 300 µl phenol/chloroform solution, pH 5.2 (Ambion, USA) was added and the mixture was vigorously vortexed. The sample was centrifuged at 12,000 *g* for 5 min. The upper aqueous layer was transferred to a new microcentrifuge tube. A total of 850 µl absolute ethanol and 30 µl of 3 M sodium acetate were added. The sample was frozen at −20°C for 1 hour in order to enhance RNA precipitation. Each sample was centrifuged at 12, 000 *g* for 20 min at 4°C to pellet the RNA. The RNA pellet was then washed with ice-cold 70% ethanol and air dried. Subsequently, the dried RNA was dissolved in 50 µl of RNAse free ddH_2_O. The RNA quantization was conducted using the Nanodrop analyzer. The integrity of RNA was determined using Bioanalyzer (Algilent, USA). The RNA samples with RIN value of 10 were as of high quality and were chosen to proceed to the next step.

These RNA samples were subjected to poly(A) polymerization using Poly(A) Tailing Kit purchased from Epicentre (USA). The reaction volume was 50 µl and it contained 25 µg of RNA, 1 mM ATP, 4 U Poly(A) polymerase and 1 X reaction buffer. The reaction was conducted at 37°C for 30 min. The (A)-tailed RNA samples were subjected to phenol/chloroform extraction according to the same method as mentioned above. The RNA's concentration was determined using Nanodrop analyzer. The resultant purified A-tailed RNA samples were then subjected to microarray analysis.

The Agilent microarray platform using the 8×15 k microarray format was used in this study. Each slide could accommodate 8 independent samples and each single compartment contained approximately 15, 000 oligonucleotide probes representing all 5721 ORFs annotated by Holden et al. (2004) [9]. Each probe was 60 nucleotides long. The design was kindly provided by Prof. Sheila Nathan (Universiti Kebangsaan Malaysia) and was custom-made by Agilent Technologies (USA) (Probe ID: 019078). The probe detail is available at NCBI GEO database with accession number GPL13233.

The One-Color Microarray-Based Gene Expression Analysis (Low input Quick Amp Labelling Kit) provided by Agilent Technologies (USA) was used in this study. All the reagents and chemicals were included in the kit. The manufacturer's instruction was strictly followed without any modification. A total of 200 ng poly(A)-tailed RNA sample was used as the starting amount. Briefly, the RNA sample was reverse transcribed to cDNA using reverse transcriptase AffinityScript and T7 promoter primer. The resultant cDNA was then subjected to *in vitro* transcriptional amplification using T7 RNA polymerase in which Cyanine 3-CTP was incorporated into the resultant cRNA. The resultant cRNA sample was purified using Absolute RNA Nanoprep Kit (Stratagene, USA).

The purified cRNA was quantitated using NanoDrop ND-1000 UV-VIS Spectrophotometer (Thermo Scientific, USA) to determine the cRNA yield and the Cyanine 3 labelling efficiency. A total of 600 ng resultant cRNA was subjected to enzymatic fragmentation in a total reaction volume of 25 µl. The fragmented cRNA was then added with 25 µl 2X hybridization buffer. A volume of 40 µl was subjected to hybridization with probe slide at 65°C for 17 hours. After performing the recommended washing steps, the slide was then scanned using Agilent Microarray Scanner with Green Dye channel and 5 µm of scan resolution. The resultant high resolution images were then subjected to data extraction using Agilent Feature Extraction Software. The extracted gene expression data was later analyzed using GeneSpring GX software (Agilent Technologies, USA).

The raw expression data of six samples were adjusted to a threshold value of 1, a median shift normalization value of 75 percentile and a baseline transformation using median of all samples. Three filtered data of each strain were group together to compare the gene expression of the wild type and versus mutant strains. The differentially expressed genes of both strains were obtained after entity list was filtered at p-value P< = 0.05 (T Test unpaired). The fold change of corresponding gene was expressed as log_2_ values. A fold change value of 2.0 and above was considered as differentially expressed genes.

### Real-time PCR validation of microarray results

The same RNA samples used for microarray study were subjected to real time PCR analysis in order to validate the microarray result. The one step iScript One-Step RT-PCR Kit With SYBR Green (BioRad, USA) was used in real time PCR. The real time thermal cycler used was BioRad CFX (BioRad, USA). A total of ten genes (Table S1B) were chosen for validation. Normalization was performed against housekeeping gene BPSL2758 (*glyA*) which encodes serine hydroxymethyltransferase [11], [12]. The real time PCR ingredient of 25 µl final volume contained 1X SYBR Green RT-PCR reaction mixture, 300 nM of each primer, 200 ng of RNA template, 1 µl of iScript reverse transcriptase; the final volume was 50 µl. The thermal steps of PCR used are of manufacturer's recommendations. A standard curve was constructed to determine the PCR efficiency (R) of each gene. To do this, a 2 fold serial dilution of the RNA template was used and the same PCR condition was applied. The real-time RT-PCR result was analyzed using Bio-Rad CFX manager (BioRad, USA). This quantization experiment was performed in triplicate and the manufacturer's general guidelines were followed. The fold change values (log_2_) values of all genes obtained from real-time quantification and microarray study were plotted. The calculated r value (slope) was used for evaluation of RNA microarray output data. The value of 1 represents an ideal correlation between RNA microarray and real-time PCR quantification.

### Oxidative stress sensitivity assay

The ΔBPSS1356 mutant showed reduced production of cytochrome bd respiratory oxidase. The oxidative stress sensitivity assay was thus performed to characterize the effect of oxidative stress on wild type and mutant strains. The disc inhibition assay was executed as described by Tunpiboonsak *et al.* (2010) [13]. Briefly, a few isolated colonies of both strains were individually inoculated into a 5 ml sterile saline solution until the turbidity of cell suspension was equivalent to 0.5 McFarland standard. A sterile cotton swab was used to spread the cells to the entire surface of LB agar plates evenly. The plates were left to dry at room temperature. Subsequently, 6 mm paper discs containing 10 µl of 0%, 2.5%, 5.0%, 10%, 15%, 20%, 25%, 30% and 35% hydrogen peroxide were placed on the cell lawn. All LB agar plates were incubated overnight at 37°C. Growth inhibition zones were measured after 24 hours incubation. Each strain was studied in triplicates.

### Growth pattern of *B. pseudomallei* ΔBPSS1356 mutant in minimal medium with glycerol as sole carbon source

The absence of BPSS1356 in *B. pseudomallei* down-regulated the glycerol metabolism related genes as indicated by the result of microarray experiment. Thus, the growth kinetics of both strains, grown using glycerol as the sole carbon source, were determined. The parameters used were the same as in the study for growth pattern of wild type and ΔBPSS1356 mutant as described earlier. The medium used was M9 minimal medium supplemented with 0.2% glycerol. The optical density values were recorded at 12-hour intervals for up to 108 hours.

### Growth pattern of *B. pseudomallei* ΔBPSS1356 mutant in high salt medium

Based on the microarray results, the BPSS1356 gene is believed to be involved in the regulation of ion transportation. Therefore, *B. pseudomallei* wild type and ΔBPSS1356 mutant were subjected to a high-salt condition to determine their growth kinetics. Pumirat et al. (2010) [14] reported that the growth of *B. pseudomallei* was slightly attenuated when LB supplemented with 320 mM NaCl medium was used. The parameters used were the same as in the study for growth pattern of wild type and ΔBPSS1356 mutant as described earlier. The optical density values were recorded every 2 hour interval for up to 30 hours.

### Osmotic stress assay

The osmotic stress assay method was performed according to Subsin et al. (2003) [15] for wild type *B. pseudomallei* K96243 and ΔBPSS1356 mutant. The bacterial cultures were prepared by overnight growth in LB broth at 37°C with rotation at 180 rpm. Bacterial cells were then harvested, washed and resuspended in M9 medium supplemented with 4 M NaCl. The resuspended cells were incubated at 37°C with shaking at 180 rpm. The colony forming unit (c.f.u.) per ml values were calculated by plating diluted cell suspensions on LA agar plates after 0, 12, 18 and 24-hour intervals. The osmotic stress assay for each strain was performed in triplicates. The viable counts were expressed as the percentage of survival after osmotic shock.

## Result

### The interactome of RpoC-N

**Figure 1:**
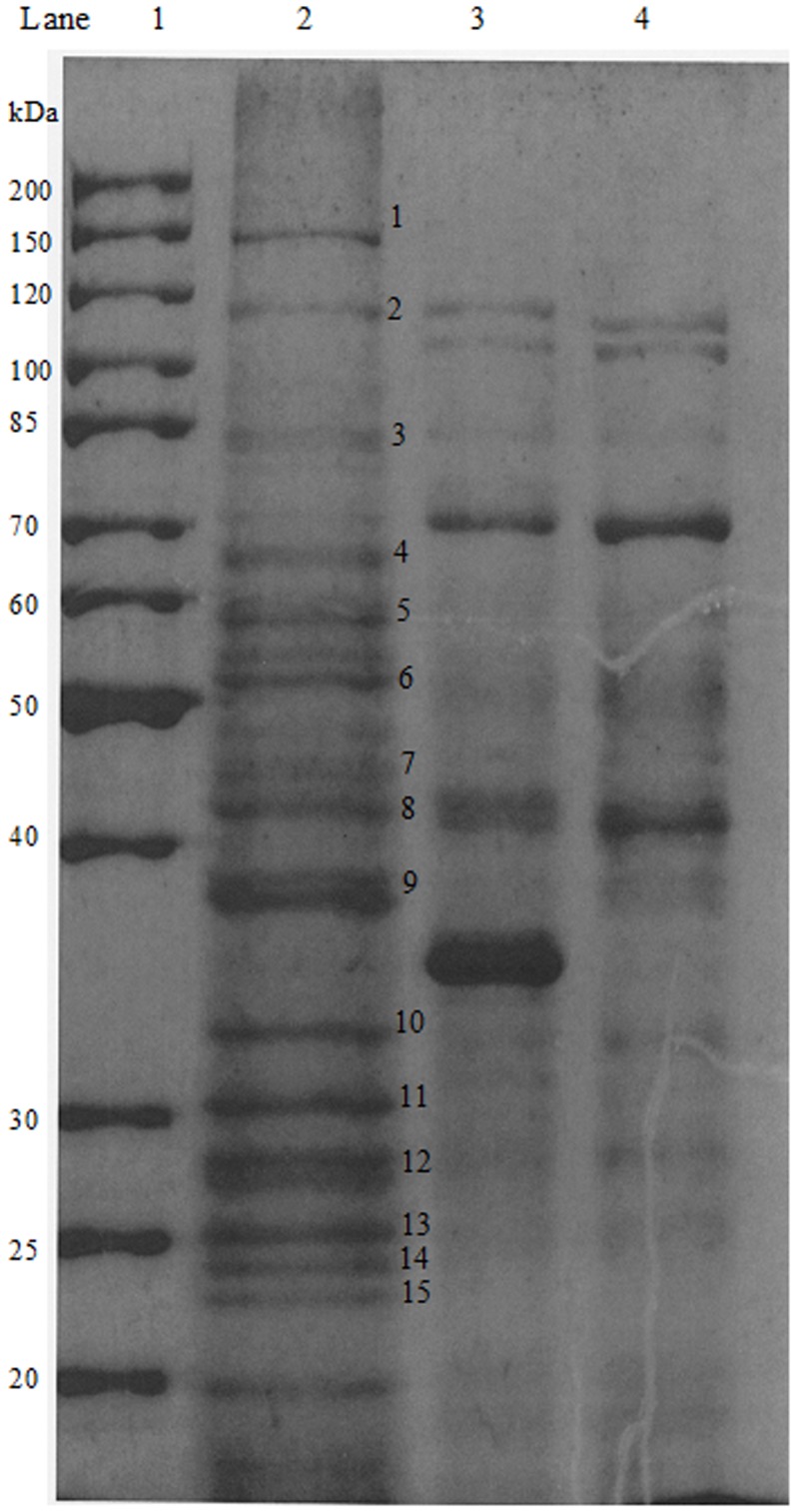
The SDS-PAGE analysis of pull down assay. Lane 1: Protein Ladder. Lane 2: First elution sample that subjected to Maldi-TOF; Lane 3: Second elution; Lane 4: Negative control. A total of 15 gel slices from Lane 2 were excised for MALDI-TOF analysis. The protein identities of the numbered locations in lane 2 are shown in Table 1.

A total of 15 discernible protein bands were chosen for Maldi-TOF analysis and the identities of the proteins obtained using Mascot search are as listed in Table 1. Intriguingly, the BPSS1356 appeared as two isoforms with sizes of approximately 80 kDa and 120 kDa (Figure 1, Band 2 and Band 3 of Lane 2). BPSS1356 is also an uncharacterized conserved protein that found only in *Burkholderia* genus. The identities of four protein bands failed to be determined by Maldi-TOF analysis. This could due to peptide degradation during the sample preparation.

**Table 1:** Identities of RpoC-N interactive proteins with locus tags and their COG annotations.

Protein band	Locus Tag	COG Annotation (Accession|Name[Category]), Calculated Mw
1	BPSL3221	COG0085|RpoB, DNA-directed RNA polymerase, beta subunit/140 kD subunit [Transcription], 153 kDa
2	BPSS1356	COG3246|COG3246, Uncharacterized conserved protein [Function unknown], 125 kDa
3	BPSS1356	COG3246|COG3246, Uncharacterized conserved protein [Function unknown], 125 kDa
4	BPSL2515	COG0539|RpsA, Ribosomal protein S1 [Translation, ribosomal structure and biogenesis], 62 kDa
5	N/A	(Failed to be identified by Maldi-TOF analysis)
6	N/A	(Failed to be identified by Maldi-TOF analysis)
7	N/A	(Failed to be identified by Maldi-TOF analysis)
8a	BPSL1246	COG2197|CitB, Response regulator containing a CheY-like receiver domain and an HTH DNA-binding domain [Signal transduction mechanisms/Transcription], 45 kDa
8b	BPSL2270	COG0148|Eno, Enolase [Carbohydrate transport and metabolism], 46 kDa
9a	BPSL2116	COG1064|AdhP, Zn-dependent alcohol dehydrogenases [General function prediction only], 36 kDa
9b	BPSL3187	COG4266|Alc, Allantoicase [Nucleotide transport and metabolism], 37 kDa
9c	BPSS1944	COG0202|RpoA, DNA-directed RNA polymerase, alpha subunit/40 kD subunit [Transcription], 36 kDa
10	BPSL3207	COG0092|RpsC, Ribosomal protein S3 [Translation, ribosomal structure and biogenesis], 30 kDa
11	N/A	(Failed to be identified by Maldi-TOF analysis)
12	BPSL3224	COG0081|RplA, Ribosomal protein L1 [Translation, ribosomal structure and biogenesis], 24 kDa
13	BPSL3188	COG0522|RpsD, Ribosomal protein S4 and related proteins [Translation, ribosomal structure and biogenesis], 23 kDa
14	BPSS0213	Hypothetical protein, 22 kDa
15	BPSL1403	COG0740|ClpP, Protease subunit of ATP-dependent Clp proteases [Posttranslational modification, protein turnover, chaperones/Intracellular trafficking and secretion], 24 kDa

### Generation of *Burkholderia pseudomallei* ΔBPSS1356 mutant

**Figure 2:**
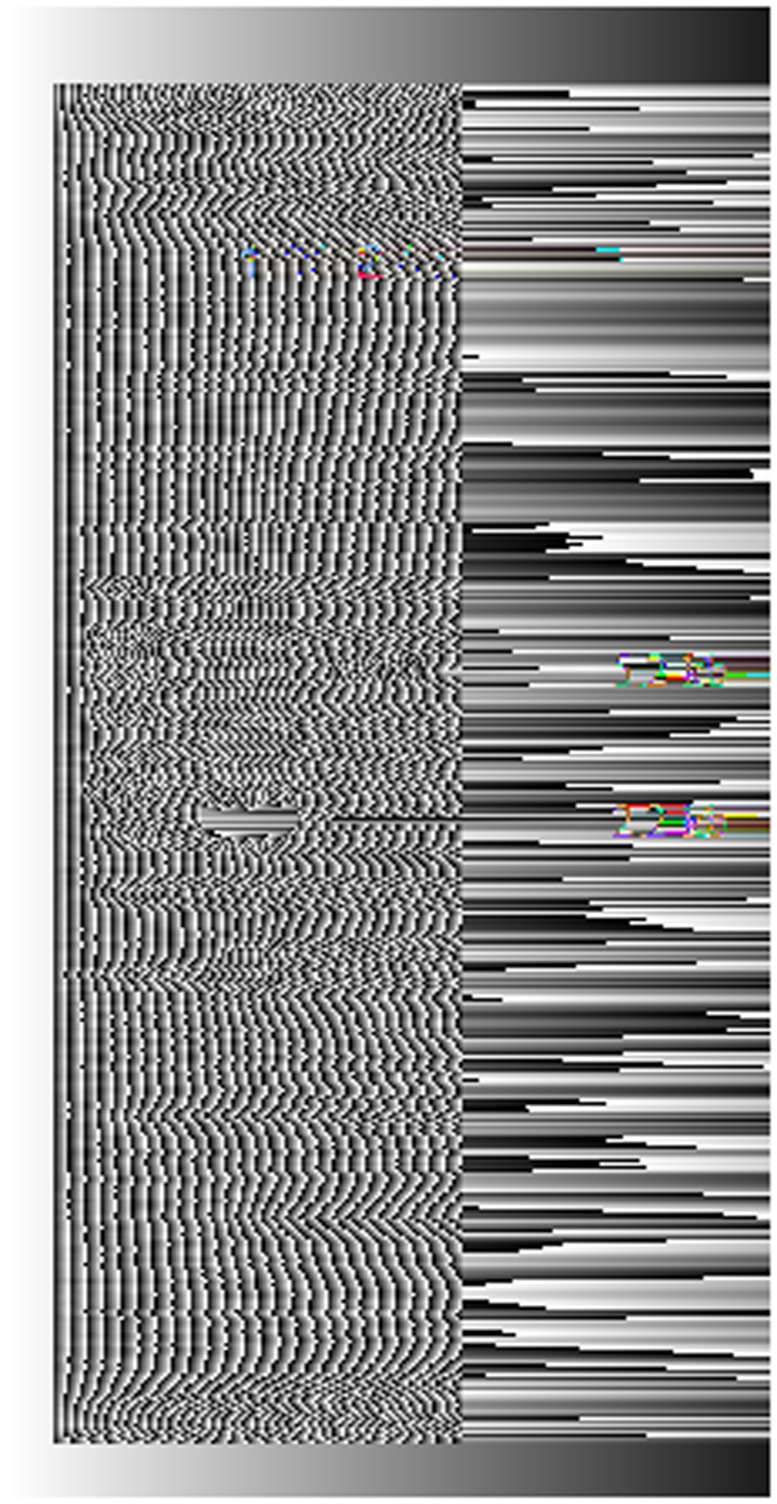
PCR screening result of mutant candidates. The PCR amplicon of 2.2(Lane 2). This indicates that mutant (Lane 1) was obtained.

PCR amplification using outer primers was used to verify the deletion of the BPSS1356 gene in the mutant strain. The mutant resulted PCR amplicon of 2.2 kb long and the wild type gave a 5.2 kb amplicon, respectively as shown in Figure 2. The automated DNA sequencing result of this PCR amplicon confirmed the deletion of BPSS1356 gene.

### Growth curve

**Figure 3:**
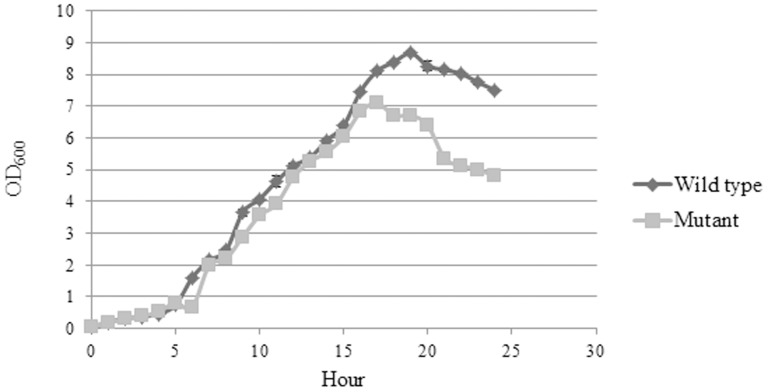
Growth rate of wild type *B. pseudomallei* K96243 and ΔBPSS1356 mutant strains in LB broth medium. Mutant strain exhibited less cell density during stationary growth phase.

When LB broth was used as the growth medium, both wild type *B. pseudomallei* K96243 and ΔBPSS1356 mutant showed the same growth rate from the lag to the early stationary phases (Figure 3). However, the ΔBPSS1356 mutant showed a greater decrement rate of cell density in stationary phase as compared to wild type. After about 16 hours of incubation, the cell density of the mutant strain culture started to decrease. This could possibly due to cell lysis progressing at a higher rate as compared to the wild type. This suggested that BPSS1356 might play a role in maintaining cell integrity during the stationary growth phase. However, both strains demonstrated no difference in growth rate when M9 minimal medium was used (data not shown).

### Electron microscopy

**Figure 4:**
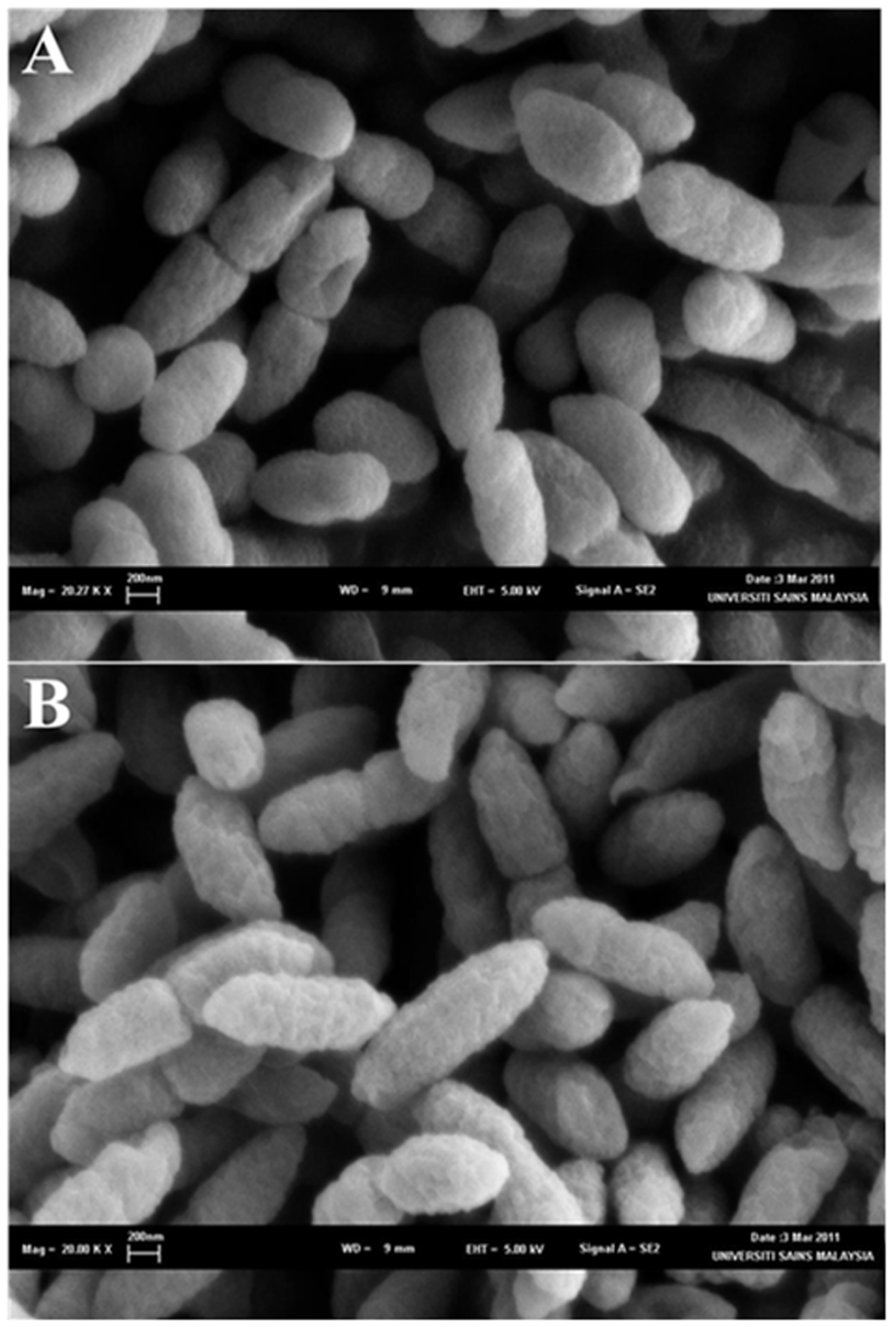
Scanning electron microscopy of (A) wild type *B. pseudomallei* and (B) ΔBPSS1356 mutant strains. Mutant strain showed rougher cell surface.

**Figure 5:**
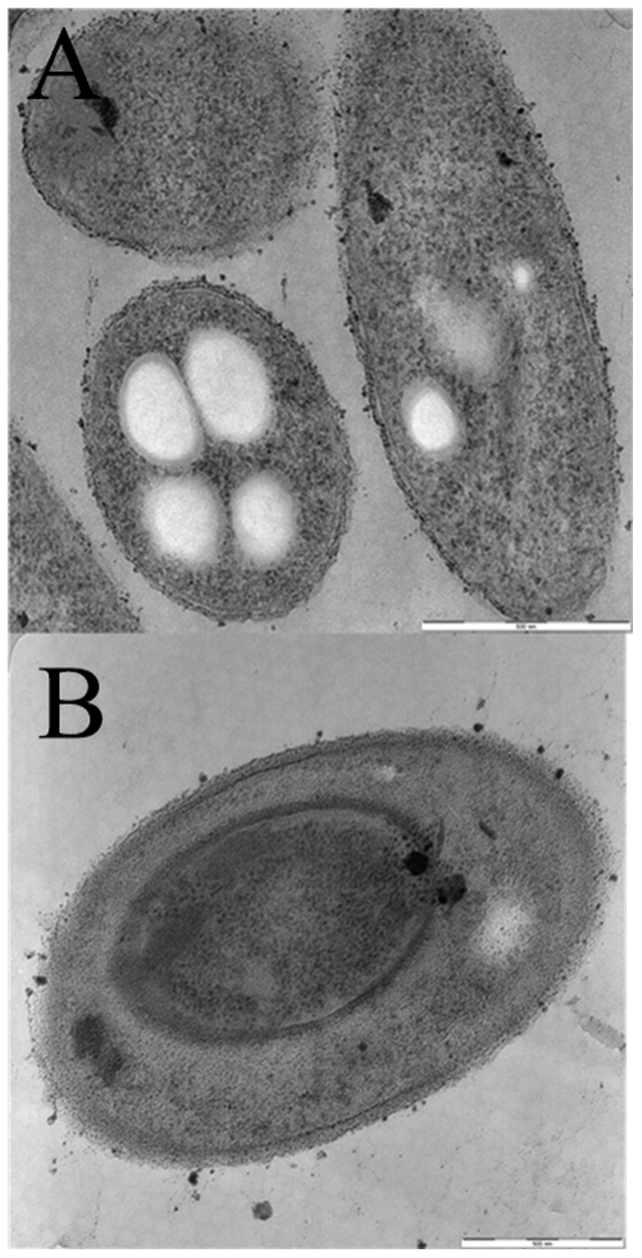
Transmission electron microscopy of (A) wild type *B. pseudomallei* and (B) ΔBPSS1356 mutant strains. Mutant showed shrunken cytoplasm.

The cells' surface of the ΔBPSS1356 mutant showed no difference with the wild type in terms of shape, width and length when examined using SEM (Figure 4). However, the cells of mutant strain exhibited a rougher cell surface than the wild type. The rough surface architecture of the mutant cells could possibly be due to less tolerance to the SEM preparation steps. TEM was also performed to examine the interior of the ΔBPSS1356 mutant and was compared with the wild type. As judged from Figure 5, the mutant showed an interesting effect of shrunken cytoplasm compartment and an expansion of periplasmic space. This appearance looked similar to bacterial plasmolysis when exposed to hypertonic solvent [16]. This observation suggested that BPSS1356 play a role in maintaining the osmotic balance between the inner compartment and the outer space of the cells.

### Biofilm formation assay

**Figure 6:**
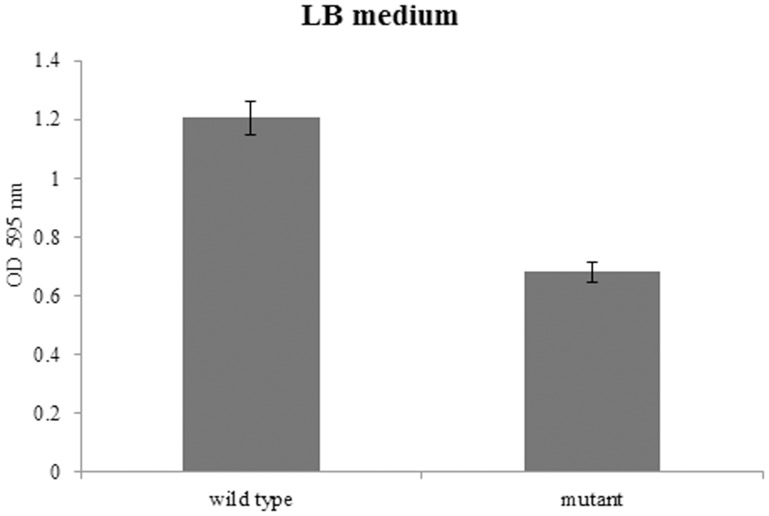
Biofilm formation analysis of wild type *B. pseudomallei* and ΔBPSS1356 mutant strains in LB. The ΔBPSS1356 mutant exhibited decreased biofilm formation.

The role of BPSS1356 in biofilm formation was investigated using the microtitre plate assay. When LB broth was used as the growth medium, ΔBPSS1356 mutant exhibited decreased biofilm formation. As shown in Figure 6, Δ1356 mutant showed a decrement of 40% (p = 0.0015; student t test) biofilm mass compared to the wild type. The decrement could due to the reduced growth rate of mutant in stationary phase. It is therefore BPSS1356 may not directly involve in biofilm formation. However, there was no significant difference between the wild type and Δ1356 mutant if M9 minimal medium was used as culture medium (data not shown).

### Global transcriptional analysis

Based on the RNA microarray analysis, the wild type *B. pseudomallei* and ΔBPSS1356 mutant showed differences in global gene expression from cultures grown in LB broth. Compare to the wild type, the mutant showed down-regulation in 303 genes and up-regulation in 289 genes when variations of more than 2 fold were considered. When a higher stringency of analysis which only considers 4 fold expression change was performed, a total of 63 genes (30 genes of Chromosome 1; 33 genes of chromosome 2) showed reduced expression in *B. pseudomallei* Δ1356 mutant. Whereas, the expression of 26 genes (21 genes of chromosome 1; 5 genes from chromosome 2) were enhanced in the mutant compared to the wild type. These differentially expressed genes were classified using COG (Clusters of Orthologous Groups) annotation system which has four functional groups: metabolism, information storage and processing, cellular processes and unknown function (Table 2). The complete list of differentially expressed genes with at least 4 fold value is shown in Table S2.

**Table 2:** Numbers of genes affected upon deletion of BPSS1356, categorized using COG functional categories annotation system.

COG classification	Number of genes down-regulated in mutant	Number of genes up-regulated in mutant
**Metabolism**		
Lipid metabolism	5	4
Energy production and conversion	5	1
Amino acids transport and metabolism	6	1
Secondary metabolites biosynthesis, transport and catabolism	4	2
Carbohydrate transport and metabolism	5	1
Coenzyme metabolism	1	1
**Cellular processes**		
Inorganic ion transport and metabolism	3	2
Signal transduction	2	1
Cell envelope biogenesis, outer membrane	1	0
Intracellular trafficking and secretion	1	0
Cell mobility and secretion	1	0
**Information storage and processing**		
Transcription	2	1
Translation, ribosomal structure and biogenesis	2	0
Nucleotide transport and metabolism	1	0
**Unknown function**		
General prediction	6	1
Function unknown	18	11
Total	63	26

Under the metabolism group, a total of 36 genes were found to be affected upon deletion of BPSS1356. These genes were further grouped into six functional categories: lipid metabolism (9 genes), energy production and conversion (6 genes), amino acid transport and metabolism (7 genes), carbohydrate transport and metabolism (6 genes), secondary metabolite biosynthesis, transport and catabolism (6 genes) and coenzyme metabolism (2 genes). Therefore, absence of BPSS1356 in *B. pseudomallei* affected the metabolism related genes most in which 40% (36 of 89 genes) were differentially expressed as compared to the wild type.

A total of 8 out of 9 genes that were related to lipid metabolism were derived from 3 putative operons (BPSL0648 to BPSL0651, BPSL1954 to BPSL1955 and BPSL0473 to BPSL0493). The adjacent genes were also considered for analysis with a fold change cut off value of 2.0. Two operons were down-regulated in ΔBPSS1356 mutant while one operon was up-regulated (Table S3). To deduce whether the lipid metabolism pathway was connected with the operons, those genes were mapped with the KEGG pathway database [17]. The result turned up to be inconclusive, as there was no specific lipid metabolism pathway which was related to the operons. This inconclusive result is due to either one gene was present in different pathways or some of the genes were not present in the lipid metabolism pathway. The lack of lipid metabolism study using *B. pseudomallei* and its related species made the analysis more challenging to perform.

In the energy production and conversion category, glycerol metabolism was affected upon removal of BPSS1356. The genes *glpK* (glycerol kinase, BPSL0687) and *glpA* (Glycerol-3-phosphate dehydrogenase, BPSL0688) were down-regulated in mutant compared to the wild type. The fold change values were 10.48 and 23.28, respectively. The gene *glpF* (glycerol uptake facilitator, BPSL0686) located immediately upstream of *glpK* was also down-regulated with a fold change value of 10.08. These three genes are possibly coregulated as a single transcript that is involved in glycerol based energy by producing dihydroxyacetone phosphate to enter the glycolysis process [18]. Besides, BPSS1356 indicated an important role in oxygen-based respiratory system of *B. pseudomallei*. The genes *cydB* (cytochrome bd oxidase subunit 2, BPSS0234) and *cydA* (cytochrome bd oxidase subunit, BPSS0235) were down-regulated in mutant with a fold change values of 6.58 and 5.77. They encoded cytochrome bd respiratory oxidase that culminates the reduction of oxygen to water [19].

BPSS1356 influences the amino acid transport and metabolism category. It could be possibly functioning as a positive regulator of arginine metabolism. In ΔBPSS1356 mutant, four consecutive genes *arcD*, *arcA*, *arcB* and *arcC* which code for arginine/ornithine antiporter (BPSL1742), arginine deiminase (BPSL1743), ornithine carbamoyltransferase (BPSL1744) and carbonate kinase (BPSL1745), respectively were markedly down-regulated (15.96, 18.68, 9.08 and 11.62). These four genes could be coregulated as single operon since they located continuously and transcribed in the same direction.

A total of 11 genes involved in cellular processes group were affected as well. A total of 5 genes belonged to the inorganic ion transport and metabolism category. The remaining 6 genes belonged to 4 other categories: signal transduction mechanism (3 genes), cell envelope biogenesis and outer membrane (1 gene), intracellular trafficking and secretion (1 gene) and cell motility and secretion (1 gene). A minimal effect was observed on the cell information storage and processing cluster with total of only 6 genes affected: 3 genes corresponded to transcription; 2 genes corresponded to translation, ribosomal structure and biogenesis and 1 gene corresponded to nucleotide transport and metabolism.

In the inorganic ion transport and metabolism category, BPSS1433 was down-regulated in the ΔBPSS1356 mutant. It is a member of an arsenic resistance locus which comprises *arsR* (transcriptional regulator, BPSS1430), BPSS1431 (unknown function), *arsC* (arsenate reductase, BPSS1432) and *arsD* (arsenite effux pump BPSS1433). All these genes were significantly down-regulated with fold change values of 4.83, 7.30, 14.92 and 14.27, respectively. In the COG annotation system, these genes were annotation with different names. However, the genes' affiliations were changed to *ars* to avoid confusion based on their sequence homology with arsenic resistance related genes [20]. In the same category, the BPSS0766 genes presumably encodes chloride channel protein (EriC) was severely suppressed in the mutant with a fold change value of 32.99 It indicated that BPSS1356 could functioned as major regulatory factor in the ion transportation process. Cell secretion system was affected as BPSS1613, BPSS1614, BPSS1617 and BPSS168 were down-regulated at least four fold. These are members of Type III secretion system (cluster 2). This locus consisted of BPSS1613 to BPSS1629 (16 ORFs). A total of 10 ORFs were found down-regulated at least 2 fold as well (list not shown). A large number of poorly annotated genes were also differentially expressed between the wild type and ΔBPSS1356 mutant. A total of 36 genes from general prediction category (7 genes) and function unknown category (29 genes) were observed. The general prediction category comprises protein members with predicted biochemistry activity but different function. The proteins that have no evidence of known functions are classified in the unknown category. The BPSL0324 was the most heavily suppressed gene with a fold change value of 42.87. This protein was classified in general prediction category. It shared homology with ubiquitous sodium bile acid symporter indicating that BPSS1356 might somehow play a critical regulation role in ion transportation.

### Real-time PCR validation

**Figure 7:**
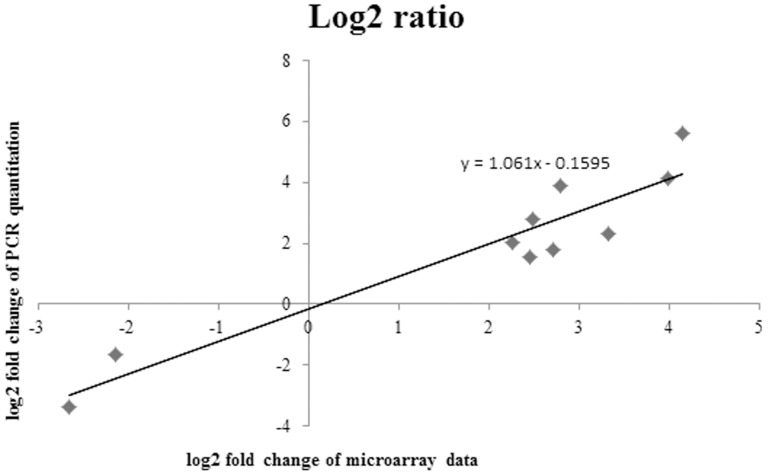
Log_2_ fold change ratio of real time PCR quantization versus microarray. The slope value of 1.06 represents the both RNA quantification methods were in congruent to each other.

The standard curves of all selected and reference genes were constructed to obtain the value of PCR efficiency (R). The R value was generated automatically by the Bio-Rad CFX manager software. The R values are listed in Table S4. The normalized fold change value of a transcript when comparing wild type over mutant is expressed as Nw∧ Nm = R^Cq(m)^ ∧ R^Cq(w)^ X R^Cq(rm)^ ∧ R^Cq(rw)^ (N represents the initial mRNA copy number, w represents wild type, m represent mutant, r represent reference gene, R represents value of PCR efficiency, Cq represents cycle value of quantification). The fold change values of each chosen candidate are also listed in Table S4. The fold change values (log_2_) values of all genes obtained from real-time quantification and microarray study were plotted. The calculated r value (slope) was 1.06 and this showed that the resultant data obtained from both methods of RNA quantification are in good agreement (Figure 7). This showed that the RNA microarray output data closely represented the actual transciptomic status of the cells.

### Oxidative stress sensitivity assay

**Figure 8:**
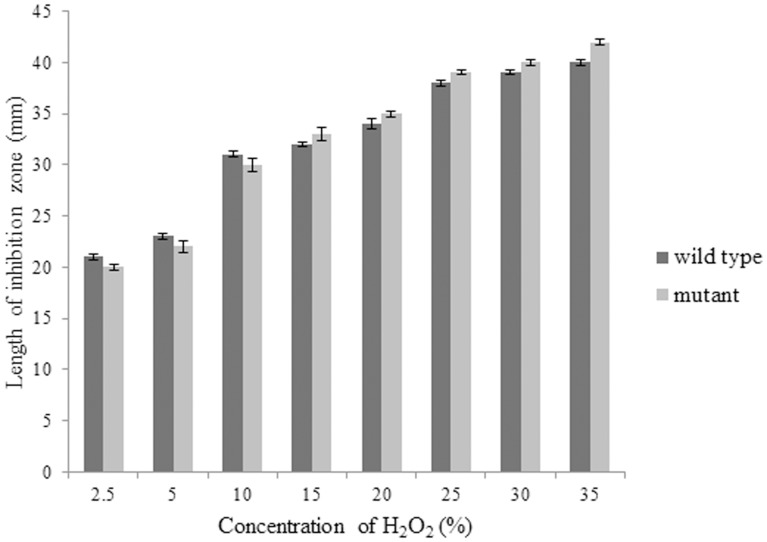
Oxidative stress assay of wild type *B. pseudomallei* and ΔBPSS1356 mutant strains. Both strains showed no significant difference in toleration to oxidative stress.

The results for this assay are as shown in Figure 8. The wild type and mutant strains showed no significant difference in response to various concentrations of hydrogen peroxide. This indicates that the down-regulation of cytochrome bd respiratory oxidase does not increase the sensitivity of the mutant cells towards oxidative stress.

### Growth pattern of ΔBPSS1356 mutant in minimal medium with glycerol as sole carbon source

**Figure 9:**
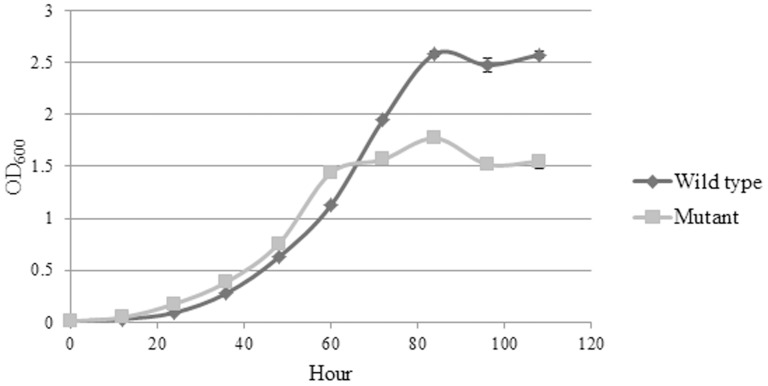
Growth kinetics of wild type *B. pseudomallei* and ΔBPSS1356 mutant strains when grown using glycerol as the sole carbon source. The ΔBPSS1356 mutant strains showed greater aggregation after 60 hours of incubation.

The ΔBPSS1356 mutant showed a slightly higher growth rate in the first 60 hours incubation as shown in Figure 9. Both the wild type and ΔBPSS1356 mutant strains showed cell aggregation during the incubation. The aggregation appeared as insoluble pellet that settled at the bottom of the cultures. However, the level of cell aggregation was markedly greater for ΔBPSS1356 mutant cells after 60 hours of incubation. The mutant culture showed reduced planktonic cell mass as compared to the wild type. This observation demonstrated that BPSS1356 is involved in glycerol metabolism as deduced from the microarray analysis.

### The growth pattern of ΔBPSS1356 mutant in high salt medium

**Figure 10:**
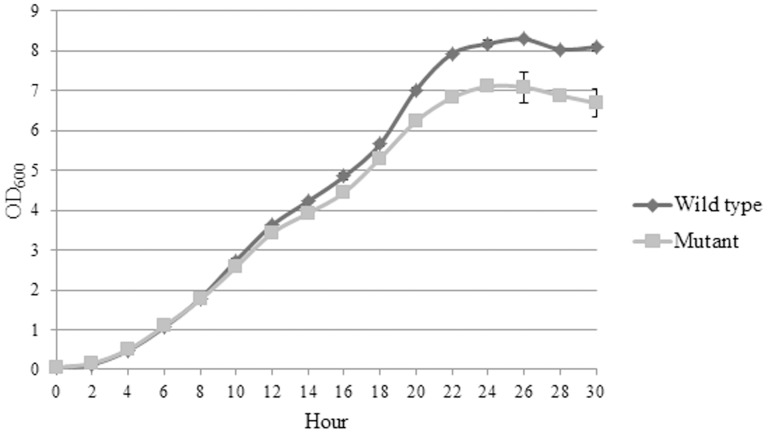
Growth kinetics of wild type *B. pseudomallei* and ΔBPSS1356 mutant strains when grown using high salt medium. The ΔBPSS1356 mutant showed a reduced growth compared to the wild type after 8 hours of incubation.

The growth patterns of both strains in high salt medium are shown in Figure 10. Both the wild type *B. pseudomallei* K96243 and ΔBPSS1356 mutant strains showed the same growth rate for the first 8 hours after inoculation. Subsequently, the ΔBPSS1356 mutant showed a reduced growth compared to the wild type. The ΔBPSS1356 mutant exhibited less cell mass than wild type when the plateau phase was achieved. This different pattern of post stationary growth could be attributed to the regulatory role of BPSS1356 in the ion transportation genes as shown by the microarray study. The retardation of ion intake could possibly be the cause of reduced fitness of ΔBPSS1356 mutant.

### Osmotic stress assay

The survival rates of wild type and ΔBPSS1356 mutant were similar (data not shown). This result indicates that the lost of BPSS1356 gene did not result in any significant changes in response to hyper osmotic shock although the cell fitness of ΔBPSS1356 mutant was compromised when it was grown using high salt medium.

## Discussion

*B. pseudomallei* is the causal agent of melioidosis, a severe disease that is endemic in tropical areas of Southeast Asia and Northern Australia [21]. The bacterium has a variety of biological repertoire that enables it to invade the immune system. A search for novel virulent factors and genetic adaptations that allow this bacterium survive intracellularly within its host has been greatly facilitated by the availability of the complete genome sequence of *B. pseudomallei*. However, more basic studies on *B. pseudomallei* are still needed to enable it to become a successful model for studying invasion and dissection.

The core unit of bacterial RNA polymerase comprises four different type of subunits (α_2_ββ′ω). Another subunit called sigma factor (σ) binds to the core enzyme to form the holoenzyme. The RNA polymerase bound sigma factor confers promoter recognition and melting [22]. The minimal region encompassing the promoter and RNA polymerase complex consisted of a small portion of the N-terminal region of the β′ subunit and a partial segment of the σ factor [6]. This assembly serves as a model to study the mechanism of transcription initiation. In this study, we used the N-terminal domain of the RNA polymerase β′ subunit (referred to as RpoC-N from here on) from *B. pseudomallei* as the bait in a pull-down experiment to isolate and identify protein partners that may also contribute to the transcriptional process in this bacterium.

The pull-down assay bound RNA polymerase subunits (RpoA and RpoB) indicating that the RpoC-N His-tagged at the N-terminus was possibly folded to enable its anticipated partners to attach to it. This also showed the effectiveness of the pull-down assay. Amongst the pulled down proteins, four were ribosomal subunits (RpsA, RpsC, RplA and RpsD) which are involved in translation. This observation is in good agreement with the result reported by Butland et al. (2005) [5] who showed that there were direct protein interactions between RNA polymerase and ribosomal proteins. Arifuzzaman et al. (2006) [4] reported the same observation as well using the recombinantly produced RpoC as the bait in their pull-down experiment. Proshkin et al. (2010) [23] showed that RNA polymerase and ribosome work in partnership during gene expression. The interactions involved were presumed to engage direct protein-protein dockings which resulted in a giant enzymatic complex [24]. In this study, the pull-down assay revealed several proteins which interacted with RpoC-N including a gene product of a hypothetical gene BPSS1356.

Based on the genome annotation of Holden et al. (2004) [9], approximately 25% of *B. pseudomallei* total genes were found to be hypothetical. This category of genes does not have any known homologs when compared to other species. It is unclear whether they encode actual proteins. Based on the early annotation, BPSS1356 was one of the members of this category. From this study, BPSS1356 was found to be a real protein coding gene and its protein interacted with RNA polymerase. This relatively large protein (125 kDa) is present only in the *Burkholderia* genus based on the tBlastN analysis. The fact that it does not exist in non *Burkholderia* bacteria indicates that it is probably not an essential gene. Amongst the 59 strains of *B. pseudomallei* with genome information available in NCBI, BPSS1356 is found in 58 strains. The tBlastN analysis revealed that BPSS1356 is highly conserved with only a maximum variation of 4 amino acids found amongst its homologs. Of interesting note, the absence of BPSS1356 in strain 1258a could be due to the melioidosis patient was infected by more than one strain of *B. pseudomallei*, this is supported by the observation that the relapse strain 1258 b possesses BPSS1356 [25]. Thus, BPSS1356 could possibly assist in the development of melioidosis or the dormancy of *B. pseudomallei* in its infected host.

The start codon of BPSS1356 was verified by integrating an in-frame C-terminal His-Tag coding sequence into the chromosomal copy of BPSS1356 [26]. The C-terminal His-tagged BPSS1356 protein produced by resultant strain was then purified and subjected to an N-terminal sequencing analysis which subsequently revealed its start codon. The validated start codon was identical to the *in silico* prediction. Moreover, the success of BPSS1356 purification under native condition suggests that it is localized in cytoplasm. This observation contradicts to a finding that BPSS1356 is present in outer membrane fraction [27]. Thus, the localization of BPSS1356 requires further validation.

An isogenic BPSS1356 deletion strain (ΔBPSS1356) was constructed in order to understand the biological role of this hypothetical gene by observing phenotypic differences between this mutant and the wild type. To provide insight into the factors affecting the observed shifts in physiology, a comparative transcriptomic microarray analysis was also performed between the mutant and wild type strains. The expression levels of 63 genes were significantly down-regulated and 26 up-regulated in the mutant for at least 4 fold change.

Both ΔBPSS1356 and wild type strains displayed similar growth rate when grown using Luria Bertani (LB) broth and defined minimal medium (M9). However, the ΔBPSS1356 mutant exhibited a greater rate of decline in cell density during stationary phase as compared to the wild type in LB broth. Based on the microarray analysis, a possible cause for cell mass reduction might be the result of reduced production of cytochrome bd respiratory oxidase which is encoded by *cydB* (BPSS0234) and *cydA* (BPSS0235). This postulation was made based on the phenotype of a cytochrome bd oxidase minus mutant of *Corynebacterium glutamicum* which exhibited reduced cell mass during stationary growth phase [28]. In the case of *C. glutamicum*, the *cydAB* deletion mutant showed a similar trend during exponential phase compared to the wild type strain when glucose minimal medium was the growth medium. The mutant exhibited 40% reduction of cell biomass compared to wild type in stationary phase [28]. However, the reduced expression of cytochrome bd in ΔBPSS1356 mutant did not increase the sensitivity of the mutant to low oxygen tension. The transcriptional reduction in the mutant might not be strong enough to produce a discernible change in the cellular phenotype. Cytochrome bd is a respiratory reductase found commonly in various prokaryotes which culminates the reduction of oxygen to water [19]. In *E. coli*, it has been shown to be an integral component of the cytoplasmic membrane [29]. Its expression was induced by various environmental stresses such as low oxygen tension, alkalization of the medium, high temperature, presence of cyanide and high hydrostatic pressure (reviewed in [19]). Cytochrome bd respiratory oxygen reductase was also stipulated to be an important virulence factor of various aerobic pathogens that can survive within microaerobic environment upon host invasion: *Mycobacterium tuberculosis* in mouse lung [30], *Brucella suis* in macrophage cells [31], *Pseudomonas aeruginosa* in host lung [32] and a closely related species *Burkholderia cenocepacia* in lung during long term residency [33].

The microarray result showed that *arcD* (arginine/ornithine antiporter, BPSL1742), *arcA* (BPSL1743), *arcB* (BPSL1744) and *arcC* (BPSL1745) were found greatly down-regulated in the mutant strain with fold change values of 15.96, 18.68, 9.08 and 11.62, respectively. The arginine deiminase system (ADS) catalyzes the conversion of arginine to ornithine, ammonia and carbon dioxide with production of ATP. It comprises three major enzymes which are ArcA (arginine deiminase), ArcB (ornithine carbamoyl transferase) and ArcC (carbamate kinase) [34]. Chantratita et al. (2011) [35] characterized the proteomic profiles of Type I (wrinkled) and Type III (smooth) and showed that two enzymes of the arginine deiminase system (ArcA and ArcC) were enhanced in the later. A similar result was also observed when the proteomic profile of *B. pseudomallei* isolated from relapsing melioidosis (Type III colony morphology) was compared to its counterpart (Type I) obtained during primary infection [36]. All three major enzymes of ADS were found to be up-regulated in bacterial cells of the smooth colony (relapse) compared to the wrinkled colony (primary). However, the colony morphology of ΔBPSS1356 mutant appeared to be the same with wild type when grown on Ashdown agar (data not shown).

The ADS was found to have minimal influence on the virulence of *B. pseudomallei*. An ADS deletion mutant of *B. pseudomallei* did not become avirulent when infection studies were performed using either macrophage cell lines [35] and murine models [37]. However, in the wild type *B. pseudomallei*, the ADS genes (*arcA*, *arcB* and *arcC*) gave a higher expression level during a long term residency in its host [36]. The same observation was also obtained in *Pseudomonas aeruginosa* [38] suggesting that the ADS plays an important role in long term adaptation within an infected host.

The ADS is also involved in other forms of adaptations. It has also been shown to be a key factor in acid tolerance in *B. pseudomallei* [35] as well as other bacteria such as *Streptococcus suis* [39], *Listeria monocytogenes* [40], *Streptococcus pyogenes* [41] and oral *streptococci* [42]. In addition, in a mouse model experiment, the ADS of *L. monocytogenes* was found to be a virulent factor [40]. It was needed to maintain the bacterial survival within the macrophage phagosome. The *arcA*, *arcB* and *arcC* genes were induced in the presence of arginine, acidic condition and low oxygen tension [35], [41], [43], [44]. Down-regulation of the ADS expression upon deletion of BPSS1356 indicated that the latter could presumably act as a transcriptional factor for ADS-associated cellular response. Interestingly, both ADS and cytochrome bd respiratory oxygen reductase related genes were enhanced during oxygen limited environment. This indicated the possibility of BPSS1356 serving as a common positive regulator of a subset of genes which are responsive to limited oxygen supply.

The regulatory role of BPSS1356 is also reflected in glycerol metabolism. Glycerol related energy production genes consist of *glpF* (glycerol uptake facilitator, BPSL0686), *glpK* (glycerol kinase, BPSL0687) and *glpA* (Glycerol-3-phosphate dehydrogenase, BPSL0688) and they are located side by side in the same transcriptional direction. Their expressions were severely suppressed in ΔBPSS1356 mutant with fold change values of 10.08, 10.48 and 23.28, respectively. The down-regulation of the glycerol related genes suggests that glycerol assimilation and metabolism are controlled by BPSS1356. The ΔBPSS1356 mutant cells tend to aggregate when glycerol was used as the carbon source when compared to the wild type. This observation links the role of BPSS1356 in glycerol metabolism. GlpF is the well characterized membrane-bound aquaporin which allows transportation of glycerol and water through the cell membrane [45]. GlpK is a kinase that conducts the phosphorylation of glycerol to produce glycerol-3-phosphate (G3P). The resultant G3P is later oxidized by either G3P dehydrogenase or G3P oxidase and both enzymes produce dihydroxyacetone phosphate which enters the glycolytic pathway [18]. In *E. coli*, the anaerobic form of G3P dehydrogenase (GlpA) generates NADH_2_ whereas the aerobic form of G3P oxidase (GlpD) generates hydrogen peroxide. Both enzymes share high similarity in their protein primary sequences [18]. When the *B. pseudomallei* BPSL0688 amino acid sequence was aligned with the *E. coli* K12 protein reference sequences, the result revealed that BPSL0688 matches GlpD better than GlpA with identities of 52% and 28%, respectively. This alignment result strongly suggests that BPSL0688 gene is the homologue of the *E. coli glpD*. If it is truly *glpA*, the corresponding operon members *glpBC* should also be present as in the case of *E. coli*. GlpABC forms the heterotrimeric enzyme G3P dehydrogenase (anaerobic enzyme of G3P dehydrogenase) [46]. Thus, labelling it as *glpA* as suggested by the COG annotation system is a misannotation. It is suggested that BPSL0688 is annotated as *glpD* to avoid confusion.

Glycerol metabolism was never thoroughly investigated in *B. pseudomallei*. In *Mycoplasma pneumoniae*, glycerol metabolism results in the production of hydrogen peroxide, which is crucial for infection in eukaryotes. GlpD performs the formation of hydrogen peroxide which has an important cytotoxic effect against the eukaryotic cells [47]. In *Borrelia burgdorferi*, the *glpFAD* operon was required for normal fitness during the tick phase of the enzootic cycle [48]. The *glpD* mutant of this bacterium replicates at a slower rate in tick compared to the wild type although the mutant has no significant contribution to virulence in a murine infection model. Glycerol was denoted as an important carbohydrate source for glycolysis during the tick phase of the infectious cycle [48]. In *B. pseudomallei*, a deeper investigation is needed on its glycerol metabolism in order to elucidate the biological role of the *glp* operon in this pathogen.

The type III secretion system (T3SS) is an important bacterial protein secretion vehicle. In many Gram negative pathogens, it is responsible for delivery of secreted proteins directly into host cytosol [49]. *B. pseudomallei* has three T3SS clusters. The T3SS cluster 1 (T3SS1) and T3SS cluster 2 (T3SS2) were found to play a minimal role in virulence in a hamster infection model [50]. In contrast, the T3SS cluster 3 (T3SS3) is a well known virulence determinant in pathogenesis [3], [51]. The T3SS2 cluster showed high similarity with *Xanthomanas spp* suggesting that *B. pseudomallei* could also be a potential plant pathogen [52]. Lee et al. (2010) [53] tested this hypothesis by infecting tomato plant with *B. pseudomallei* and the study showed that T3SS2 was required for infection although the actual mechanism is unknown. In the *B. pseudomallei* K96243 genome, the T3SS2 proteins are encoded by the genes BPSS1613 to BPSS1629 [52]. In this study, the genes BPSS1613 to BPSS1618 and BPSS1622 to BPSS1626 were down-regulated suggesting that BPSS1356 plays a role in the expression of the genes of the T3SS2 proteins.

In *B. pseudomallei* K92643 genome revealed the existence of an arsenic resistance operon. This putative operon encodes the homologs of a transcriptional regulator (ArsR; BPSS1430), a hypothetical protein of unknown function (BPSS1431), arsenate reductase (ArsC; BPSS1432) and expulsion pump of arsenite (ArsD; BPSS1433), respectively. This putative operon was down-regulated in ΔBPSS1356 mutant when compared with the wild type. In *E. coli*, ArsC binds to arsenate resulting in the formation of a disulfide bond between the cystein residues and the reducing equivalents. The subsequent reduction of the disulfide bond results in arsenate reduction to arsenite and the later is extruded from the cells by ArsB [20]. Interestingly, GlpF also participates in the uptake of arsenite and a GlpF mutant resulted in an arsenite-insensitive phenotype [54]. The *glpF* expression showed down-regulation as well in ΔBPSS1356 mutant. Coincidentally, both ArsD and GlpF are transmembrane proteins.

Biofilm is a microorganismal consortium that adheres to biotic and abiotic surfaces. These aggregated cells are embedded with a matrix of extracellular polymeric substance matrix. Bacteria forms biofilm in response to nutrient limited condition and occurs during late stationary growth phase. These stages of bacterial physiological establishment involved transcriptional reprogramming of large scale of genes. The biofilm formation of bacteria allows their survival in unflavored living condition and it is a crucial adaptation strategy of various pathogens [55], [56]. The ΔBPSS1356 mutant showed decreased biofilm formation compared to the wild type. This study showed 40% reduction when using LB as growth medium. This observation could also due to the reduced fitness of the ΔBPSS1356 mutant in stationary phase. Moreover, the microarray results revealed no significant difference in the expression of biofilm related genes between the ΔBPSS1356 mutant and wild type strains. Additionally, the RNA samples were isolated from the cells that were undergoing exponential growth. At this stage, the expressions of genes involved in biofilm formation are not yet altered. In *B. pseudomallei*, biofilm formation purportedly did not have a direct correlation with virulence. No mortality difference was observed when mice were challenged with biofilm producing and biofilm deficient mutant strains [57]. However, Sawasdidoln et al. (2010) [58] suggested that biofilm of *B. pseudomallei* is involved in enhancing drug resistance and may be the possible cause of high relapse of melioidosis.

Bacterial ion channels are membrane proteins that responsible for the transportation of ions across membrane by flowing down their electrochemical gradient. These proteins have high selectivity of specific ions such as sodium, calcium, potassium or chloride [59]. Amongst the genes affected by the BPSS1356 deletion, the ion transportation related genes were down-regulated with the highest magnitude. The transcription of BPSL0324 (putative sodium bile acid symporter family protein) and *eriC* (chloride channel, BPSS0766) were suppressed with fold change values of 48 and 33, respectively, in ΔBPSS1356 mutant when compared to the wild type. BPSL0324 was annotated as an eukaryotic ubiquitous gene encoding a putative sodium bile acid symporter based on amino acid similarity with its eukaryotic counterparts as well as the presence of 9 transmembrane α-helical spanners [60], [61]. In eukaryotes, this transmembrane protein functions in the liver in the uptake of bile acids from portal blood plasma mediated by the co-transportation of Na^+^ [62]. A homologous ileal protein of this symporter in human is responsible for reabsorbing bile acids and taurocholate from small intestine and its mutation was characterized as the possible cause of Crohn's disease [63]. The biological function of this protein family in bacteria is yet to be determined although it is likely to play a role in Na^+^ dependent acid transportation that resides in the cell membrane [61]. The BPSL0766 contains 7 transmembrane α-helical spanners and exhibits sequence homology to the eukaryotic Cl^−^ channel (CLC) that is involved in anion transportation [64]. In *E. coli*, this family of proteins (EriC and MriT) confers survival in extreme acidic environment such as stomach acid through decarboxylation of glutamate or aspartate followed by excretion of the resulting products [65]. The EriC of *E. coli* is most likely a H^+^/Cl^−^ exchange transporter rather than a channel with a single directional flow of anion channel [66]. It has also been shown that *sycA* (homolog of *eriC*) is an essential gene in *Rhizobium tropici* CIAT899 in order to establish proficient a symbiotic relationship with its legume host. However, the molecular basis of this observation is still unknown [67].

In this study, the TEM of ΔBPSS1356 mutant displayed a shrunken cytoplasm which could be due to an expanded periplasmic space. This observation could be the consequence of NaCl intake blockage due to the suppressions of ion transportation proteins BPSS0766 and EriC. The LB medium used contained 5% NaCl, this external hyperosmotic pressure possibly triggered the exportation of H_2_O from the cytoplamic compartment of ΔBPSS1356 mutant mimicking a plasmolysis process. Bacterial plasmolysis is common during a hyperosmotic exposure and shrinkage of the cytoplasm is a typical observation [16], [68]. The most well characterized bacterial sodium channel is NaChBac from *Bacillus halodurans* [69]. This type of Na channel is not ubiquitous in bacteria and is not found in *B. pseudomallei* [70]. To date, there is no other example of bacterial sodium channel was reported. This suggests that BPSL0324 deserves further investigation to interrogate the possible role in sodium transportation vehicle. The plasmolysis of Δ1356 mutant when exposed to high salt condition would also be the probable cause of its rougher cell exterior when observed using scanning electron microscopy. The same observation was reported when *E. coli* was used as the model. The wrinkling of the *E. coli* cell wall was due to the dehydration force upon cell exposure to hyperosmotic condition [16]. The ΔBPSS1356 mutant exhibited reduced cell mass when grown in high salt medium and this could be due to the effect on the transportation of ions upon deletion of the gene BPSS1356. A NaCl sensitivity assay was performed and the result showed no viability difference between wild type and mutant (data not shown). This could be explained by the attenuation of ion transportation genes in ΔBPSS1356 mutant could not produce enough strength to cause viability discrepancy between wild type and mutant.

The upstream sequences of BPSS1356 affected genes were analyzed using Virtual Footprint V3 [71] to search for a consensus putative promoter sequence controlled by known bacterial transcriptional factors. This attempt failed to reveal any consensus motif. A genus specific and yet to be characterized transcriptional regulation might be present in *B. pseudomallei*. BPSS1356 can possibly play a role in this process.

In summary, the significantly attenuated genes in the ΔBPSS1356 mutant code for membrane proteins. These include BPSL0324 and *eriC* (Cl^−^ channel) with fold change values of 42.87 and 32.99, respectively. Transportation related genes *arcD* (arginine transportation), *glpF* (glycerol permease) and *arsD* (expulsion pump of arsenite) were down-regulated at least 10 fold as well. The plasmolysis exhibited by the ΔBPSS1356 mutant served as an evidence in the role of the gene in ion transportation. The BPSS1356 was found to be present in RpoC-N interactome. This suggested that BPSS1356 may act as a positive *Trans*-acting regulatory element in the transcriptional regulation of ion transportation related genes. This hypothetical gene BPSS1356 is most likely necessary for *B. pseudomallei* survival in a harsh environment. BPSS1356 deletion mutant also exhibited down-regulation of genes encoding cytochrome bd and arginine deiminase system. This was further supported by a reduced biofilm formation and a reduced cell mass during stationary phase. BPSS1356 has also been shown to affect transcriptional expressions of genes involved in glycerol metabolism, Type III secretion system, arsenic resistance pathway and lipid metabolism. It is therefore obvious that BPSS1356 played a role in regulation of many genes which explain its multiple regulatory effects. Transcription start sites mapping should be performed in order to precisely map the promoter regions of the affected genes and the outcome will provide a more detail on the underlying transcriptional regulation by BPSS1356. The hypothetical gene BPSL0324 merits further investigation since it is likely involved in sodium transportation via an uncharacterized machinery.

### Microarray data accession number

The raw microarray data and the normalised signal intensity values were deposited to the Gene Expression Omnibus (GEO) (http://www.ncbi.nlm.nih.gov/geo/), they are accessible through GEO series accession number GSE53710.

## Supporting Information

Table S1 **Primers used in this study.** A) List of primers used in ΔBPSS1356 mutant construction. B) List of primers used in real-time PCR validation.
(DOCX)

Table S2 **Differentially expressed genes for at least 4 fold change, sorted according to COG annotations.**
(DOCX)

Table S3 **The differentially expressed regulons of lipid metabolism.**
(DOCX)

Table S4 **Result of real time PCR validation, fold change value was calculated as Nw∧ Nm = R^Cq(m)^ ∧ R^Cq(w)^ X R^Cq(rm)^ ∧ R^Cq(rw)^.**
(DOCX)
