# Correction: The Multiple Roles of Hypothetical Gene BPSS1356 in *Burkholderia pseudomallei*

**DOI:** 10.1371/journal.pone.0103394

**Published:** 2014-07-24

**Authors:** The PLOS ONE Staff

Figure 2 became corrupted during the production process. The publisher apologizes for the error. Please see the correct Figure 2 here.

**Figure 2:**
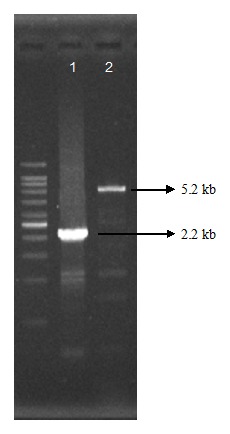
PCR screening result of mutant candidates. The PCR amplicon of 2.2(Lane 2). This indicates that mutant (Lane 1) was obtained.
