# Supplementary material for: The Multiple Roles of Hypothetical Gene BPSS1356 in Burkholderia pseudomallei
Source: PLoS One. 2014 Jun 13;9(6):e99218. doi: 10.1371/journal.pone.0099218 (PMC4057154; doi:10.1371/journal.pone.0099218)
Supplement: Table S1 — Primers used in this study. A) List of primers used in ΔBPSS1356 mutant construction. B) List of primers used in real-time PCR validation. (DOCX) [file pone.0099218.s001.docx]

Table S1.

A) List of primers used in ∆BPSS1356 mutant construction.

| Primer | Sequence 5'-3' | Underlined | Genome location |
| --- | --- | --- | --- |
| 1356USF | ATAGATCTTCGACGTAGACAACGGCAACGCA | *Bgl*II | 1852487-1852509 |
| 1356USR | AAAAATAAGCTTGGTGATGTAGAAGGTCTTGACCAT | *BamH*I | 1853417-1853440 |
| 1356DSF | AAAAATAAGCTTCTGCAGGCGTCGATGACCGACTA | *BamH*I | 1856729-1856751 |
| 1356DSR | TAGAGCTCCGCGGACTTCATGACCTTTGC | *Sac*I | 1857706-1857726 |
| NQCAT | TAACGGCAAAAGCACCGCCGGACATCA | *N/A* | *N/A* |
| NQREV | ACATGTGGAATTGTGAGCGGATAACAA | *N/A* | *N/A* |
| 1356U-out | ACCGCGCACGTGCCGTTATT | *N/A* | 1852448-1852467 |
| 1356D-out | CCACGACGTGAGCCCGTAGAA | *N/A* | 1857871-1857891 |
| 1356_mutSeq | TCGATCCGGTTTCCGGCGAAAC | *N/A* | 1853299-1853320 |

B) List of primers used in real-time PCR validation.

| Gene | Forward primer sequence; Reverse primer sequence (5’ to 3’) | Product size (bp) |
| --- | --- | --- |
| BPSL0649 | GCAGTTCCTGTGGTCGATCT; ATGTCTTCCTGCACCCACTC | 186 |
| BPSL0686 | CATACATCGCGGAGTTCATC; ACGTAGACCGCGACGAATAC | 154 |
| BPSL1062 | GGCCAAAGAAGAATTGATCG; GCCCTTTCGTGAGGTCATAG | 194 |
| BPSL1742 | TCCTGATCCCGTACTTCTGG; CGGACAGCAGCAGATACTTG | 174 |
| BPSL3091 | AACAAGAAATCGCCATGGTT; GTCGACCGTGTGCGTCAG | 152 |
| BPSS0234 | GCATCGTGTTTCCGTTCTTT; AGAGCACGGTCGAGTAGACG | 150 |
| BPSS0238 | GCTGCTCGAGCTGTACATGA; GATTGACGACCGGATTGAAC | 170 |
| BPSS1076 | CGCAATGTGACGCTGTATCT; GCCTGTCCAACCTGCATACT | 192 |
| BPSS1430 | GTCTTCCGCACGTTGGTG; GCAGCAGTTTTCCGTGAGAT | 216 |
| BPSS1638 | TATCCGGCCTGTATTTCGAG; GTCGACGAGCAGTTTCTTCC | 176 |
| BPSS2758 | GAAAACGTCCGTCAGGAAGA; TCGACGATGTCCACGTATTC | 161 |
