# Supplementary material for: The Multiple Roles of Hypothetical Gene BPSS1356 in Burkholderia pseudomallei
Source: PLoS One. 2014 Jun 13;9(6):e99218. doi: 10.1371/journal.pone.0099218 (PMC4057154; doi:10.1371/journal.pone.0099218)
Supplement: Table S2 — Differentially expressed genes for at least 4 fold change, sorted according to COG annotations. (DOCX) [file pone.0099218.s002.docx]

| Table S2. Differentially expressed genes for at least 4 fold change, sorted according to COG annotations. | Locus tag | Fold Change | Expression in wild type compared to mutant | COG Predictions (Accession\|Name[Category]) |
| --- | --- | --- | --- | --- |
|  | **Metabolism** | | | |
|  | **Lipid metabolism** | | | |
| 1 | BPSL0649 | 6.97598 | up | COG1250\|FadB, 3-hydroxyacyl-CoA dehydrogenase [Lipid metabolism]. |
| 2 | BPSL0650 | 5.420852 | up | COG0183\|PaaJ, Acetyl-CoA acetyltransferase [Lipid metabolism]. |
| 3 | BPSL0651 | 4.915419 | up | COG1024\|CaiD, Enoyl-CoA hydratase/carnithine racemase [Lipid metabolism]. |
| 4 | BPSL1954 | 4.490573 | up | COG2057\|AtoA, Acyl CoA:acetate/3-ketoacid CoA transferase, beta subunit [Lipid metabolism]. |
| 5 | BPSL1955 | 5.236005 | up | COG1788\|AtoD, Acyl CoA:acetate/3-ketoacid CoA transferase, alpha subunit [Lipid metabolism]. |
| 6 | BPSL0483 | -4.19834 | down | COG1960\|CaiA, Acyl-CoA dehydrogenases [Lipid metabolism]. |
| 7 | BPSL0485 | -4.83647 | down | COG0318\|CaiC, Acyl-CoA synthetases (AMP-forming)/AMP-acid ligases II [Lipid metabolism / Secondary metabolites biosynthesis, transport, and catabolism]. |
| 8 | BPSL0487 | -5.78964 | down | COG0332\|FabH, 3-oxoacyl-[acyl-carrier-protein]. |
| 9 | BPSS1638 | -4.38674 | down | COG2030\|MaoC, Acyl dehydratase [Lipid metabolism]. |
|  | **Energy production and conversion** | | | |
| 1 | BPSL0687 | 10.4836 | up | COG0554\|GlpK, Glycerol kinase [Energy production and conversion]. |
| 2 | BPSL0688 | 23.28282 | up | COG0578\|GlpA, Glycerol-3-phosphate dehydrogenase [Energy production and conversion]. |
| 3 | BPSL2188 | 4.370341 | up | COG2224\|AceA, Isocitrate lyase [Energy production and conversion]. |
| 4 | BPSS0234 | 6.582971 | up | COG1294\|AppB, Cytochrome bd-type quinol oxidase, subunit 2 [Energy production and conversion]. |
| 5 | BPSS0235 | 5.768292 | up | COG1271\|CydA, Cytochrome bd-type quinol oxidase, subunit 1 [Energy production and conversion]. |
| 6 | BPSL3420 | -5.01481 | down | COG1012\|PutA, NAD-dependent aldehyde dehydrogenases [Energy production and conversion]. |

|  | **Amino acid transport and metabolism** | | | |
| --- | --- | --- | --- | --- |
| 1 | BPSL1742 | 15.95647 | up | COG0531\|PotE, Amino acid transporters [Amino acid transport and metabolism]. |
| 2 | BPSL1743 | 18.68118 | up | COG2235\|ArcA, Arginine deiminase [Amino acid transport and metabolism]. |
| 3 | BPSL1744 | 9.084441 | up | COG0078\|ArgF, Ornithine carbamoyltransferase [Amino acid transport and metabolism]. |
| 4 | BPSL1745 | 11.62009 | up | COG0549\|ArcC, Carbamate kinase [Amino acid transport and metabolism]. |
| 5 | BPSS0840 | 6.370133 | up | COG1063\|Tdh, Threonine dehydrogenase and related Zn-dependent dehydrogenases [Amino acid transport and metabolism / General function prediction only]. |
| 6 | BPSS1563 | 4.688636 | up | COG1280\|RhtB, Putative threonine efflux protein [Amino acid transport and metabolism]. |
| 7 | BPSL0486 | -5.75092 | down | COG0019\|LysA, Diaminopimelate decarboxylase [Amino acid transport and metabolism]. |
|  | **Carbohydrate transport and metabolism** | | | |
| 1 | BPSL0686 | 10.0847 | up | COG0580\|GlpF, Glycerol uptake facilitator and related permeases (Major Intrinsic Protein Family) [Carbohydrate transport and metabolism]. |
| 2 | BPSL0827 | 4.086415 | up | COG0524\|RbsK, Sugar kinases, ribokinase family [Carbohydrate transport and metabolism]. |
| 3 | BPSL2740 | 4.278459 | up | COG2814\|AraJ, Arabinose efflux permease [Carbohydrate transport and metabolism]. |
| 4 | BPSS0762 | 5.416431 | up | COG2271\|UhpC, Sugar phosphate permease [Carbohydrate transport and metabolism]. |
| 5 | BPSS0965 | 16.2588 | up | COG2140\|COG2140, Thermophilic glucose-6-phosphate isomerase and related metalloenzymes [Carbohydrate transport and metabolism / General function prediction only]. |
| 6 | BPSL0601 | -4.45789 | down | COG0235\|AraD, Ribulose-5-phosphate 4-epimerase and related epimerases and aldolases [Carbohydrate transport and metabolism]. |
|  | **Secondary metabolites biosynthesis, transport, and catabolism** | | | |
| 1 | BPSL2738 | 4.993526 | up | COG0179\|MhpD, 2-keto-4-pentenoate hydratase/2-oxohepta-3-ene-1,7-dioic acid hydratase (catechol pathway) [Secondary metabolites biosynthesis, transport, and catabolism]. |
| 2 | BPSL2739 | 4.929629 | up | COG3508\|HmgA, Homogentisate 1,2-dioxygenase [Secondary metabolites biosynthesis, transport, and catabolism]. |
| 3 | BPSS0586 | 22.12733 | up | COG1020\|EntF, Non-ribosomal peptide synthetase modules and related proteins [Secondary metabolites biosynthesis, transport, and catabolism]. |
| 4 | BPSS0764 | 4.142786 | up | COG1028\|FabG, Dehydrogenases with different specificities (related to short-chain alcohol dehydrogenases) [Secondary metabolites biosynthesis, transport, and catabolism / General function prediction only]. |
| 5 | BPSL1778 | -5.54633 | down | COG1020\|EntF, Non-ribosomal peptide synthetase modules and related proteins [Secondary metabolites biosynthesis, transport, and catabolism]. |
| 6 | BPSS0439 | -4.72532 | down | COG1335\|PncA, Amidases related to nicotinamidase [Secondary metabolites biosynthesis, transport, and catabolism]. |
|  | Coenzyme metabolism | | |  |
| 1 | BPSL2623 | 4.789172 | up | COG0001\|HemL, Glutamate-1-semialdehyde aminotransferase [Coenzyme metabolism]. |
| 2 | BPSL1768 | -5.9111 | down | COG1429\|CobN, Cobalamin biosynthesis protein CobN and related Mg-chelatases [Coenzyme metabolism]. |
|  | **Cellular processes** | | | |
|  | **Inorganic ion transport and metabolism** | | | |
| 1 | BPSS0766 | 32.99226 | up | COG0038\|EriC, Chloride channel protein EriC [Inorganic ion transport and metabolism]. |
| 2 | BPSS1433 | 14.27239 | up | COG0798\|ACR3, Arsenite efflux pump ACR3 and related permeases [Inorganic ion transport and metabolism]. |
| 3 | BPSL2377 | 11.71621 | up | COG2703\|COG2703, Hemerythrin [Inorganic ion transport and metabolism]. |
| 4 | BPSL1172 | -6.05748 | down | COG2216\|KdpB, High-affinity K+ transport system, ATPase chain B [Inorganic ion transport and metabolism]. |
| 5 | BPSL1783 | -7.26784 | down | COG0609\|FepD, ABC-type Fe3+-siderophore transport system, permease component [Inorganic ion transport and metabolism]. |
|  | **Signal Transduction mechanisms** | | | |
| 1 | BPSL0732 | 4.278491 | up | COG0642\|BaeS, Signal transduction histidine kinase [Signal transduction mechanisms]. |
| 2 | BPSS1432 | 14.91997 | up | COG0394\|Wzb, Protein-tyrosine-phosphatase [Signal transduction mechanisms]. |
| 3 | BPSL0597 | -6.05441 | down | COG0515\|SPS1, Serine/threonine protein kinase [General function prediction only / Signal transduction mechanisms / Transcription / DNA replication, recombination, and repair]. |
|  | **Cell envelope biogenesis, outer membrane** | | | |
| 1 | BPSS0238 | 5.488813 | up | COG0744\|MrcB, Membrane carboxypeptidase (penicillin-binding protein) [Cell envelope biogenesis, outer membrane]. |

|  | **Intracellular trafficking and secretion** | | | |
| --- | --- | --- | --- | --- |
| 1 | BPSS1617 | 4.285374 | up | COG4790\|EscR, Type III secretory pathway, component EscR [Intracellular trafficking and secretion]. |
|  | **Cell motility and secretion** | | | |
| 1 | BPSS1618 | 4.142117 | up | COG1886\|FliN, Flagellar motor switch/type III secretory pathway protein [Cell motility and secretion / Intracellular trafficking and secretion]. |
|  | **Information storage and processing** | | | |
|  | **Transcription** | | | |
| 1 | BPSS1430 | 4.825717 | up | COG0640\|ArsR, Predicted transcriptional regulators [Transcription]. |
| 2 | BPSS1610 | 7.260607 | up | COG2207\|AraC, AraC-type DNA-binding domain-containing proteins [Transcription]. |
| 3 | BPSS1467 | -7.91799 | down | COG1167\|ARO8, Transcriptional regulators containing a DNA-binding HTH domain and an aminotransferase domain (MocR family) and their eukaryotic orthologs [Transcription / Amino acid transport and metabolism]. |
|  | **Translation, ribosomal structure and biogenesis** | | | |
| 1 | BPSL1062 | 17.76 | up | COG0361\|InfA, Translation initiation factor 1 (IF-1) [Translation, ribosomal structure and biogenesis]. |
| 2 | BPSS1364a | 4.664618 | up | COG0828\|RpsU, Ribosomal protein S21 [Translation, ribosomal structure and biogenesis]. |
|  | **Necleotide transport and metabolism** | | | |
| 1 | BPSS2282 | 4.288496 | up | COG0209\|NrdA, Ribonucleotide reductase, alpha subunit [Nucleotide transport and metabolism]. |
|  | **Unknown** | | | |
|  | **General fuction prediction** | | | |
| 1 | BPSL0324 | 42.87086 | up | COG0385\|COG0385, Predicted Na+-dependent transporter [General function prediction only]. |
| 2 | BPSL0448 | 11.07542 | up | COG2110\|COG2110, Predicted phosphatase homologous to the C-terminal domain of histone macroH2A1 [General function prediction only]. |
| 3 | BPSL2415 | 4.700173 | up | COG2153\|ElaA, Predicted acyltransferase [General function prediction only]. |
| 4 | BPSS1122 | 4.223229 | up | COG2319\|COG2319, FOG: WD40 repeat [General function prediction only]. |
| 5 | BPSS1137 | 4.382299 | up | COG0679\|COG0679, Predicted permeases [General function prediction only]. |
| 6 | BPSS2286 | 8.61339 | up | COG0517\|COG0517, FOG: CBS domain [General function prediction only]. |
| 7 | BPSL1782 | -5.03461 | down | COG4114\|FhuF, Uncharacterized Fe-S protein [General function prediction only]. |

|  | **Function unknown** | | | |
| --- | --- | --- | --- | --- |
| 1 | BPSL0369 | 5.634107 | up | N/A |
| 2 | BPSL0735 | 8.714549 | up | N/A |
| 3 | BPSL0938A | 5.264895 | up | N/A |
| 4 | BPSL1063 | 15.82505 | up | N/A |
| 5 | BPSL3090 | 6.125939 | up | N/A |
| 6 | BPSL3091 | 5.608823 | up | N/A |
| 7 | BPSS0019 | 9.585729 | up | N/A |
| 8 | BPSS0239 | 5.682032 | up | COG2860\|COG2860, Predicted membrane protein [Function unknown]. |
| 9 | BPSS0763 | 5.086459 | up | COG3246\|COG3246, Uncharacterized conserved protein [Function unknown]. |
| 10 | BPSS0740 | 5.502253 | up | N/A |
| 11 | BPSS0804 | 10.3466 | up | N/A |
| 12 | BPSS1335 | 5.005026 | up | N/A |
| 13 | BPSS1431 | 7.298734 | up | N/A |
| 14 | BPSS1606 | 6.13043 | up | N/A |
| 15 | BPSS1607 | 6.431471 | up | N/A |
| 16 | BPSS1611 | 6.001973 | up | N/A |
| 17 | BPSS1613 | 7.199105 | up | N/A |
| 18 | BPSS1614 | 9.019429 | up | N/A |
| 19 | BPSL0488 | -4.6255 | down | N/A |
| 20 | BPSL0491 | -4.75381 | down | N/A |
| 21 | BPSL0492 | -4.53206 | down | N/A |
| 22 | BPSL1785 | -5.63716 | down | N/A |
| 23 | BPSL2572 | -4.13295 | down | N/A |
| 24 | BPSL2574 | -4.73005 | down | N/A |
| 25 | BPSL2576 | -4.33381 | down | N/A |
| 26 | BPSL2578 | -5.27978 | down | N/A |
| 27 | BPSL2920 | -4.26186 | down | N/A |
| 28 | BPSS1076 | -6.31558 | down | N/A |
| 29 | BPSS1462 | -4.17966 | down | COG5591\|COG5591, Uncharacterized conserved protein [Function unknown]. |
