# Supplementary material for: The Multiple Roles of Hypothetical Gene BPSS1356 in Burkholderia pseudomallei
Source: PLoS One. 2014 Jun 13;9(6):e99218. doi: 10.1371/journal.pone.0099218 (PMC4057154; doi:10.1371/journal.pone.0099218)
Supplement: Table S3 — The differentially expressed regulons of lipid metabolism. (DOCX) [file pone.0099218.s003.docx]

| Locus Tag | Fold Change | Expression in mutant | COG Predictions (Accession\|Name[Category]) |
| --- | --- | --- | --- |
| **Regulon 1** | | | |
| BPSL0648 | -2.43704 | Down | COG1960\|CaiA, Acyl-CoA dehydrogenases [Lipid metabolism]. |
| BPSL0649 | -6.97598 | Down | COG1250\|FadB, 3-hydroxyacyl-CoA dehydrogenase [Lipid metabolism]. |
| BPSL0650 | -5.420852 | Down | COG0183\|PaaJ, Acetyl-CoA acetyltransferase [Lipid metabolism]. |
| BPSL0651 | -4.915419 | Down | COG1024\|CaiD, Enoyl-CoA hydratase/carnithine racemase [Lipid metabolism]. |
| **Regulon 2** | | | |
| BPSL1954 | -4.490573 | Down | COG2057\|AtoA, Acyl CoA:acetate/3-ketoacid CoA transferase, beta subunit [Lipid metabolism]. |
| BPSL1955 | -5.236005 | Down | COG1788\|AtoD, Acyl CoA:acetate/3-ketoacid CoA transferase, alpha subunit [Lipid metabolism]. |
| **Regulon 3** | | | |
| BPSL0473 | 2.25865 | Up |  |
| BPSL0474 | 2.3175 | Up | COG2814\|AraJ, Arabinose efflux permease [Carbohydrate transport and metabolism]. |
| BPSL0475 | 3.50067 | Up | COG3239\|DesA, Fatty acid desaturase [Lipid metabolism]. |
| BPSL0477 | 3.85465 | Up | N/A |
| BPSL0478 | 2.86805 | Up | COG0842\|COG0842, ABC-type multidrug transport system, permease component [Defense mechanisms]. |
| BPSL0479 | 3.11848 | Up | COG1131\|CcmA, ABC-type multidrug transport system, ATPase component [Defense mechanisms]. |
| BPSL0480 | 2.67371 | Up | COG2175\|TauD, Probable taurine catabolism dioxygenase [Secondary metabolites biosynthesis, transport, and catabolism]. |
| BPSL0481 | 3.33486 | Up | COG2226\|UbiE, Methylase involved in ubiquinone/menaquinone biosynthesis [Coenzyme metabolism]. |
| BPSL0482 | 3.9255 | Up | COG0372\|GltA, Citrate synthase [Energy production and conversion]. |
| BPSL0483 | 4.19834 | Up | COG1960\|CaiA, Acyl-CoA dehydrogenases [Lipid metabolism]. |

Table S3. The differentially expressed regulons of lipid metabolism.

| BPSL0484 | 3.99462 | Up | COG0332\|FabH, 3-oxoacyl-[acyl-carrier-protein]. |
| --- | --- | --- | --- |
| BPSL0485 | 4.83647 | Up | COG0318\|CaiC, Acyl-CoA synthetases (AMP-forming)/AMP-acid ligases II [Lipid metabolism / Secondary metabolites biosynthesis, transport, and catabolism]. |
| BPSL0486 | 5.75092 | Up | COG0019\|LysA, Diaminopimelate decarboxylase [Amino acid transport and metabolism]. |
| BPSL0487 | 5.78964 | Up | COG0332\|FabH, 3-oxoacyl-[acyl-carrier-protein]. |
| BPSL0488 | 4.6255 | Up | N/A |
| BPSL0489 | 3.19794 | Up | N/A |
| BPSL0490 | 3.66518 | Up | N/A |
| BPSL0491 | 4.75381 | Up | N/A |
| BPSL0492 | 4.53206 | Up | N/A |
| BPSL0493 | 2.41808 | Up | COG0318\|CaiC, Acyl-CoA synthetases (AMP-forming)/AMP-acid ligases II [Lipid metabolism / Secondary metabolites biosynthesis, transport, and catabolism]. |
