# Supplementary material for: The Multiple Roles of Hypothetical Gene BPSS1356 in Burkholderia pseudomallei
Source: PLoS One. 2014 Jun 13;9(6):e99218. doi: 10.1371/journal.pone.0099218 (PMC4057154; doi:10.1371/journal.pone.0099218)
Supplement: Table S4 — Result of real time PCR validation, fold change value was calculated as Nw∧ Nm = RCq(m) ∧ RCq(w) X RCq(rm) ∧ RCq(rw). (DOCX) [file pone.0099218.s004.docx]

Table S4. Result of real time PCR validation, fold change value was calculated as

Nw^ Nm = R^Cq(m)^ ^ R^Cq(w)^ X R^Cq(rm)^ ^ R^Cq(rw)^.

| Gene | R value | Min Cq  (Wild type) | Min Cq (Mutant) | Fold change | Log_2_ Fold change | Log_2_ Fold change of Microarray |
| --- | --- | --- | --- | --- | --- | --- |
| BPSL0649 | 1.952 | 18.19667 | 21.61333 | 14.7526 | 3.882897 | 2.8024 |
| BPSL0686 | 1.984 | 22.32 | 24.68 | 4.899103 | 2.292518 | 3.3341 |
| BPSL1062 | 1.978 | 20.39667 | 24.88667 | 48.08847 | 5.587619 | 4.1506 |
| BPSL1742 | 1.944 | 20.23667 | 24.67 | 17.33635 | 4.115728 | 3.9961 |
| BPSL3091 | 1.918 | 23.40333 | 25.89667 | 6.858957 | 2.777989 | 2.4877 |
| BPSS0234 | 1.910 | 23.29333 | 25.54333 | 3.397802 | 1.764602 | 2.7187 |
| BPSS0238 | 1.930 | 24.62667 | 26.29333 | 2.971681 | 1.571279 | 2.4565 |
| BPSS1076 | 1.940 | 16.22 | 12.56333 | 0.095558 | -3.38748 | -2.659 |
| BPSS1430 | 1.936 | 19.99667 | 21.98 | 4.118267 | 2.042037 | 2.2707 |
| BPSS1638 | 1.940 | 19.62 | 18.13333 | 0.322424 | -1.63297 | -2.133 |
| BPSL2758  (reference) | 1.978 | 19.94  [Cq(rw)] | 20.38333  [Cq(rm)] | N/A | N/A | N/A |
